# Associations of obesity and body shape with erythrocyte and reticulocyte parameters in the UK Biobank cohort

**DOI:** 10.1186/s12902-023-01423-1

**Published:** 2023-08-02

**Authors:** Sofia Christakoudi, Konstantinos K. Tsilidis, Evangelos Evangelou, Elio Riboli

**Affiliations:** 1 Department of Epidemiology and Biostatistics, School of Public Health, Imperial College London, St Mary’s Campus, Norfolk Place, London, W2 1PG UK; 2 Department of Inflammation Biology, School of Immunology and Microbial Sciences, King’s College London, London, UK; 3 Department of Hygiene and Epidemiology, University of Ioannina School of Medicine, Ioannina, Greece

**Keywords:** Obesity, Body shape, Waist size, Hip size, ABSI, Erythrocytes, Reticulocytes, Haemoglobin

## Abstract

**Background:**

Obesity is associated with type 2 diabetes mellitus and chronic low-grade inflammation. Although chronic inflammatory conditions and diabetes are associated with anaemia, less is known about associations of obesity and body shape, independent of each other, with erythrocyte and reticulocyte parameters.

**Methods:**

We investigated the associations of body mass index (BMI) and the allometric body shape index (ABSI) and hip index (HI), which are uncorrelated with BMI, with erythrocyte and reticulocyte parameters (all continuous, on a standard deviation (SD) scale) in UK Biobank participants without known metabolic, endocrine, or major inflammatory conditions (glycated haemoglobin HbA1c < 48 mmol/mol, C-reactive protein CRP < 10 mg/L). We examined erythrocyte count, total reticulocyte count and percent, immature reticulocyte count and fraction (IRF), haemoglobin, haematocrit, mean corpuscular haemoglobin mass (MCH) and concentration (MCHC), mean corpuscular and reticulocyte volumes (MCV, MRV), and red cell distribution width (RDW) in multivariable linear regression models. We additionally defined body shape phenotypes with dichotomised ABSI (≥ 73 women; ≥ 80 men) and HI (≥ 64 women; ≥ 49 men), including “pear” (small-ABSI-large-HI) and “apple” (large-ABSI-small-HI), and examined these in groups according to BMI (18.5–25 normal weight; 25–30 overweight; 30–45 kg/m^2^ obese).

**Results:**

In 105,853 women and 100,854 men, BMI and ABSI were associated positively with haemoglobin, haematocrit, and erythrocyte count, and more strongly with total reticulocyte count and percent, immature reticulocyte count and IRF. HI was associated inversely with all, but least with IRF. Associations were comparable in women and men. In groups according to obesity and body shape, erythrocyte count was ~ 0.6 SD higher for obese-“apple” compared to normal-weight-“pear” phenotype (SD = 0.31*10^12^/L women, SD = 0.34*10^12^/L men), total reticulocyte count was ~ 1.1 SD higher (SD = 21.1*10^9^/L women, SD = 23.6*10^9^/L men), immature reticulocyte count was ~ 1.2 SD higher (SD = 7.9*10^9^/L women, SD = 8.8*10^9^/L men), total reticulocyte percent was ~ 1.0 SD higher (SD = 0.48% women and men), and IFR was over 0.7 SD higher (SD = 5.7% women and men). BMI but not ABSI or HI was associated more weakly inversely with MCV, MRV, and MCH, but positively with MCHC in men and RDW in women.

**Conclusions:**

In obesity uncomplicated with diabetes, larger BMI and ABSI are associated with increased erythropoiesis and reticulocyte immaturity.

**Supplementary Information:**

The online version contains supplementary material available at 10.1186/s12902-023-01423-1.

## Background

Obesity is a growing epidemic, with almost half of Europeans classified as overweight or obese and a higher prevalence for older age [1]. Obesity represents a state of chronic low-grade inflammation and increases the risk of type 2 diabetes mellitus [2]. Chronic inflammatory conditions are associated with anaemia [3]. Diabetes is also associated with anaemia, not only in relation to kidney damage [4], but also when kidney function is preserved [5]. It is, therefore, reasonable to expect that obesity is associated with lower haemoglobin and erythrocyte count, but the available information is limited, originates from smaller-scale studies with contradictory findings, and is considered inconclusive [6]. Although a meta-analysis of 26 cross-sectional and case–control studies indicated that higher body mass index (BMI) is associated with iron deficiency [7], implying an underlying iron-deficiency anaemia, the findings were unequivocal in children and adolescents but not in adults and a systematic review has highlighted higher haemoglobin concentration in obesity [8]. Little is also known about associations with waist and hip size independent of BMI, because body shape has traditionally been evaluated with waist and hip circumferences [9], which are correlated strongly positively with BMI and reflect general obesity in addition to body shape [10]. In contrast, the allometric body shape index (ABSI) and hip index (HI) reflect waist and hip sizes, correspondingly, among individuals with the same weight and height and are uncorrelated with BMI, thus permitting evaluation of body shape independent of general obesity [11, 12]. Body shape is of interest because the metabolic complications of obesity are associated unfavourably with large waist size but favourably with large hip size [13, 14].

In this cross-sectional study, using BMI, ABSI, and HI, we investigated the associations of obesity and body shape, independent of each other, with erythrocyte and reticulocyte parameters in the UK Biobank cohort, separately in women and men and in subgroups according to menopausal status in women. To identify associations more likely determined by obesity and not by comorbidities such as diabetes or inflammation, we used a subset of healthy participants without known metabolic, endocrine, or inflammatory conditions. To account for differences in body composition between BMI categories [10], we explored heterogeneity of the associations with body shape according to BMI. We hypothesised that obesity is associated with lower erythrocyte count and haemoglobin.

## Methods

### Study population

UK Biobank is a population-based cohort including half a million participants from England, Scotland, and Wales, aged 40 to 70 years at recruitment (between 2006 and 2010) and resident within 40 km of an assessment centre [15]. Consistent with our previous study on metabolic and inflammatory biomarkers [13], we included only participants with self-reported white ancestry, due to the limited number of participants from other ethnicities, precluding adequate investigation of ethnic differences. We excluded participants with a mismatch between genetically determined and self-reported sex, missing or extreme anthropometric measurements, and pregnant women. We additionally excluded participants with medical conditions, which could influence obesity and body shape or haematological and biomarker measurements, thus extracting a subset of healthy UK Biobank participants. The exclusion criteria consisted of prevalent cancer at recruitment (defined as in [16]), incident cancer or death within two years after recruitment, the following self-reported conditions at recruitment: diabetes mellitus, endocrine non-cancer illness, chronic respiratory disease, heart failure, inflammatory bowel disease, liver disease, kidney failure, or self-reported use at recruitment of lipid lowering drugs, exogenous steroids, or anti-hypertensive drugs, as in [13]. For this study, we further excluded participants with self-reported haematological conditions, autoimmune or systemic conditions, glycated haemoglobin HbA1c ≥ 48 mmol/L, C-reactive protein CRP ≥ 10 mg/L, use of iron, vitamin B12 or erythropoietin containing medications, detected nucleated red blood cells, missing or extreme red blood cell measurements, or missing information for recent weight and diet changes. In total, we excluded 295,705 (58.9%) participants. Details of the exclusions are shown in Supplementary Table S1.

### Anthropometric indices

Waist circumference was measured by trained UK Biobank technicians at the natural indent or the umbilicus and hip circumference was measured at the widest point [17]. To calculate ABSI for women and men and HI for women, we used coefficients from the National Health and Nutrition Examination Survey (NHANES) [11, 12]. To calculate HI for men, however, we used coefficients previously derived from UK Biobank data [10], to avoid the inverse correlation with BMI observed for HI calculated with coefficients from NHANES [16]. (WC – waist circumference; HC – hip circumference):

$$ABSI = 1000*WC(m) *{Weight(kg)}^{-2/3}*{Height(m)}^{5/6}$$

$${HI}_{women} = HC\left(cm\right)*{Weight\left(kg\right)}^{-0.482} * {Height(kg)}^{0.310}$$

$${HI}_{men} = HC(cm) *{Weight(kg)}^{-2/5} *{Height(cm)}^{1/5}$$

$$BMI = Weight(kg) *Height(m)^{-2}$$

We used all anthropometric indices on a continuous scale, as sex-specific z-scores (value minus mean divided by standard deviation, SD). We additionally defined body shape phenotypes with an ABSI-by-HI cross-classification as “pear” – small-ABSI-large-HI, “slim” – small-ABSI-small-HI, “wide” – large-ABSI-large-HI, “apple” – large-ABSI-small-HI, using dichotomised ABSI with cut-offs close to the sex-specific medians (≥ 73 for women; ≥ 80 for men) and HI (≥ 64 for women; ≥ 49 for men), as in our previous study on body composition [10]. We further investigated heterogeneity of the associations with body shape according to BMI with a BMI-by-ABSI-by-HI cross-classification, using World Health Organisation (WHO) categories for BMI: normal weight (BMI ≥ 18.5 to < 25 kg/m^2^), overweight (BMI ≥ 25 to < 30 kg/m^2^), and obese (BMI ≥ 30 to < 45 kg/m^2^).

### Haematological measurements

Blood samples were collected between 8 am and 9 pm, irrespective of fasting status. Haematological measurements were performed within 24 h of blood draw on Beckman Coulter LH750 automated analysers, using blood collected in EDTA (ethylenediaminetetraacetic acid) vacutainers [18]. Erythrocyte (red blood cell) count (*10^12^/L), reticulocyte percent (%), and haemoglobin concentration (g/L) were measured; mean corpuscular (erythrocyte) volume (MCV, fL) and red cell distribution width (RDW, %) were derived from a histogram of erythrocyte size; haematocrit (%), mean corpuscular (erythrocyte) haemoglobin mass (MCH, pg), mean corpuscular haemoglobin concentration (MCHC, g/L), reticulocyte count (*10^9^/L), immature (high light scatter) reticulocyte count (*10^9^/L), immature reticulocyte fraction (IRF, %), and mean reticulocyte volume (MRV, fL) were calculated [18, 19]. For comparability, we calculated sex-specific z-scores for all haematological measurements.

### Statistical analysis

Statistical analyses were performed in R version 4.1.3 [20].

We calculated regression coefficients (95% confidence intervals) for each erythrocyte or reticulocyte parameter with multivariable linear regression models, using anthropometric indices as exposure variables. To investigate associations with obesity and body shape independent of each other, we used additive models combining BMI, ABSI, and HI (sex-specific z-scores) and interpreted the estimates as SD change in haematological parameters per one SD increment of the anthropometric index. To investigate associations with body shape phenotypes, we used an additive model combining body shape phenotypes (“pear”, “slim”, “wide”, “apple”) and BMI categories and interpreted the estimates for body shape as SD difference in haematological parameters compared to the reference category (“pear”). To investigate heterogeneity of the associations with body shape according to BMI, we used the BMI-by-ABSI-by-HI cross-classification, which is equivalent to an interaction model, and interpreted estimates as SD difference in haematological parameters compared to the reference category (normal-weight-“pear”). In the main analysis, we investigated separately women and men. In a secondary analysis, we investigated separately pre-menopausal and post-menopausal women, as hormonal changes after the menopause may influence the associations.

Similarly to our previous study on metabolic and inflammatory biomarkers [13], we adjusted all models for height (continuous), age at recruitment (continuous), weight change within the last year preceding recruitment (lost weight, stable weight, gained weight), smoking status (never, former occasional, former regular, current smoker), alcohol consumption (≤ 3 times/month, ≤ 4 times/week, daily), physical activity (inactive, moderately active, active), Townsend deprivation index (continuous), region of the assessment centre, time of blood collection (continuous), fasting time (continuous), use of nonsteroidal anti-inflammatory drugs (no/yes), and in women also menopausal status (pre-menopausal, post-menopausal, unknown, for analysis of women overall), HRT use (never, former, except for analysis of pre-menopausal women), oral contraceptives use (never, former), and age at the last live birth (no live births, < 30 years, ≥ 30 years). For this study, we additionally adjusted for major dietary change in the last 5 years (no/yes), dietary intake and use of supplements, as these may influence dietary availability and absorption of iron, including the following variables: intake of fruit, vegetables, red meat, processed meat, poultry, fish, cheese, bread, cereal, tea, and coffee (continuous), use of vitamins ACE, use of vitamin D and minerals (including calcium, zinc, magnesium), and use of multivitamins (no/yes). For definition of variables, see Supplementary Methods. As missingness for covariates was minimal (< 1%), we replaced missing values with the median sex-specific value or category.

We used Wald tests to evaluate the statistical significance of individual terms. To evaluate heterogeneity of the associations with individual obesity indices according to sex and menopausal status, we used interaction terms between BMI, ABSI, or HI (sex-specific z-scores) and sex or menopausal status, as in [21]. We used likelihood ratio tests to evaluate associations with body shape phenotypes overall and heterogeneity of the associations with body shape phenotypes according to BMI category. For details on tests for heterogeneity see Supplementary Methods and figure legends. Tests for statistical significance were two-sided. We interpreted p-values as strength of the evidence for association, considering *p* < 0.001 as evidence of association (equivalent to Bonferroni correction for 50 comparisons), and *p* < 1*10^–6^ as strong evidence (equivalent to Bonferroni correction for 50,000 comparisons). Descriptions of associations as stronger or weaker are based on the size of the regression coefficients.

In sensitivity analyses, we investigated WHO categories of BMI and sex-specific tertiles for ABSI and HI, to explore potential non-linearity. To explore the influence of mutual adjustment, we examined BMI, ABSI, and HI individually, with adjustment for covariates. To explore the influence of covariates, we compared the fully adjusted models with unadjusted models. To explore the influence of exclusions, which are likely to introduce selection bias, we examined models in the unrestricted dataset, including all participants with available anthropometric and haematological measurements, without any exclusions (general UK Biobank population), with adjustment for the covariates included in the main models, but consolidating in a single category recent major dietary change due to illness or other reasons, and consolidating former and current use in a single category (ever use) for HRT and oral contraceptives.

## Results

### Cohort characteristics

The study included 105,853 women (31,457 pre-menopausal; 64,285 post-menopausal) and 100,854 men. Men had higher BMI, ABSI, erythrocyte and reticulocyte counts, haematocrit, and haemoglobin compared to women, and were less likely to have changed diet within the last five years (Supplementary Table S2). Post-menopausal women had higher BMI and ABSI compared to pre-menopausal women, as well as a larger proportion of “wide” phenotype and higher erythrocyte count and haematocrit (Supplementary Table S2). Study participants had similar lifestyle characteristics to a previously described restricted UK Biobank dataset [21].

### Associations of anthropometric indices with erythrocyte and reticulocyte parameters

**Fig. 1:**
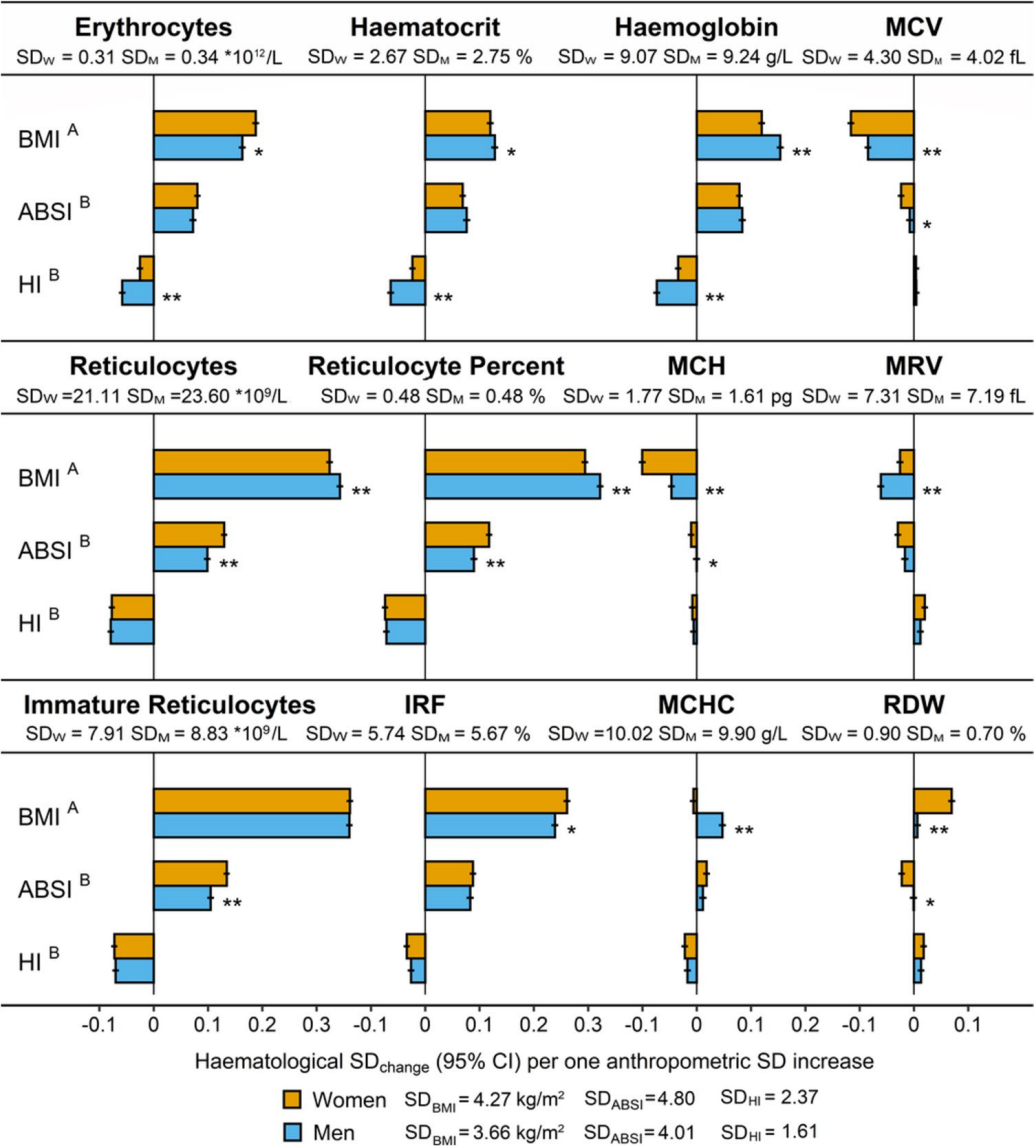
Associations of obesity and body shape indices with erythrocyte and reticulocyte parameters. ABSI – a body shape index; BMI – body mass index; CI – confidence interval; HI – hip index; IRF– immature reticulocyte fraction; MCH – mean corpuscular haemoglobin; MCHC – mean corpuscular haemoglobin concentration; MCV – mean corpuscular volume; MRV – mean reticulocyte volume; RDW – red cell distribution width; SD – standard deviation (SD_W_ – women; SD_M_ – men). SD_change_ (95% CI) – regression coefficient (95% confidence interval) interpreted as the change in haematological parameters on SD scale per one SD increase of each anthropometric index. Estimates were obtained from multivariable linear regression models, including each erythrocyte or reticulocyte parameter as an outcome variable (sex-specific z-scores, value minus mean divided by SD). Exposure variables included BMI, ABSI, and HI (sex-specific z-scores). Adjustment variables included height, age, recent weight change, smoking status, alcohol consumption, physical activity, Townsend deprivation index, dietary intake (fruit, vegetables, red meat, processed meat, poultry, fish, cheese, bread, cereals, tea, coffee), recent major dietary change, region of the assessment centre, time of blood collection, fasting time, use of nonsteroidal anti-inflammatory drugs, use of vitamins ACE, vitamin D and minerals, or multivitamins, and for women, menopausal status, hormonal replacement therapy use, oral contraceptives use, and age at the last live birth. Values are shown in Supplementary Table S3. ^A^ – p-value for the interaction term between BMI and sex, from a joint model including women (reference) and men, with adjustment for ABSI, HI, and all covariates, except female-specific, but including an interaction term between age and sex, to account for potential differences by menopausal status in women. ^B^ – *p*-value for the interaction term between ABSI and sex or HI and sex, from a joint model including women (reference) and men, with adjustment for BMI and all covariates, except female-specific, but including an interaction term between age and sex. * *p* < 0.001; ** *p* < 1*10^–6^

BMI was associated positively with erythrocyte count, haematocrit, haemoglobin, total and immature reticulocyte counts, and reticulocyte proportions (reticulocyte percent and IRF), with markedly stronger associations for reticulocytes than for erythrocytes (Fig. 1). Associations with haemoglobin and reticulocyte counts were slightly stronger in men compared to women. BMI, however, was associated inversely with MCH and MCV (more strongly in women) and MRV (more strongly in men), but positively with MCHC (only in men) and RDW (only in women).

ABSI was associated positively and HI inversely with erythrocyte count, haematocrit, haemoglobin, and the total and immature reticulocyte counts and proportions but there was little evidence for associations of ABSI or HI with cellular haemoglobin content or size (Fig. 1). Associations with ABSI were largely similar in women and men, but with HI were weaker in women for erythrocyte count, haematocrit, and haemoglobin, and were weaker in both women and men for IRF.

Associations with anthropometric indices were similar in pre-menopausal and post-menopausal women, except for a stronger positive association of ABSI with haematocrit and haemoglobin in post-menopausal women (Supplementary Figure S1).

### Association of body shape phenotypes with erythrocyte and reticulocyte parameters

**Fig. 2:**
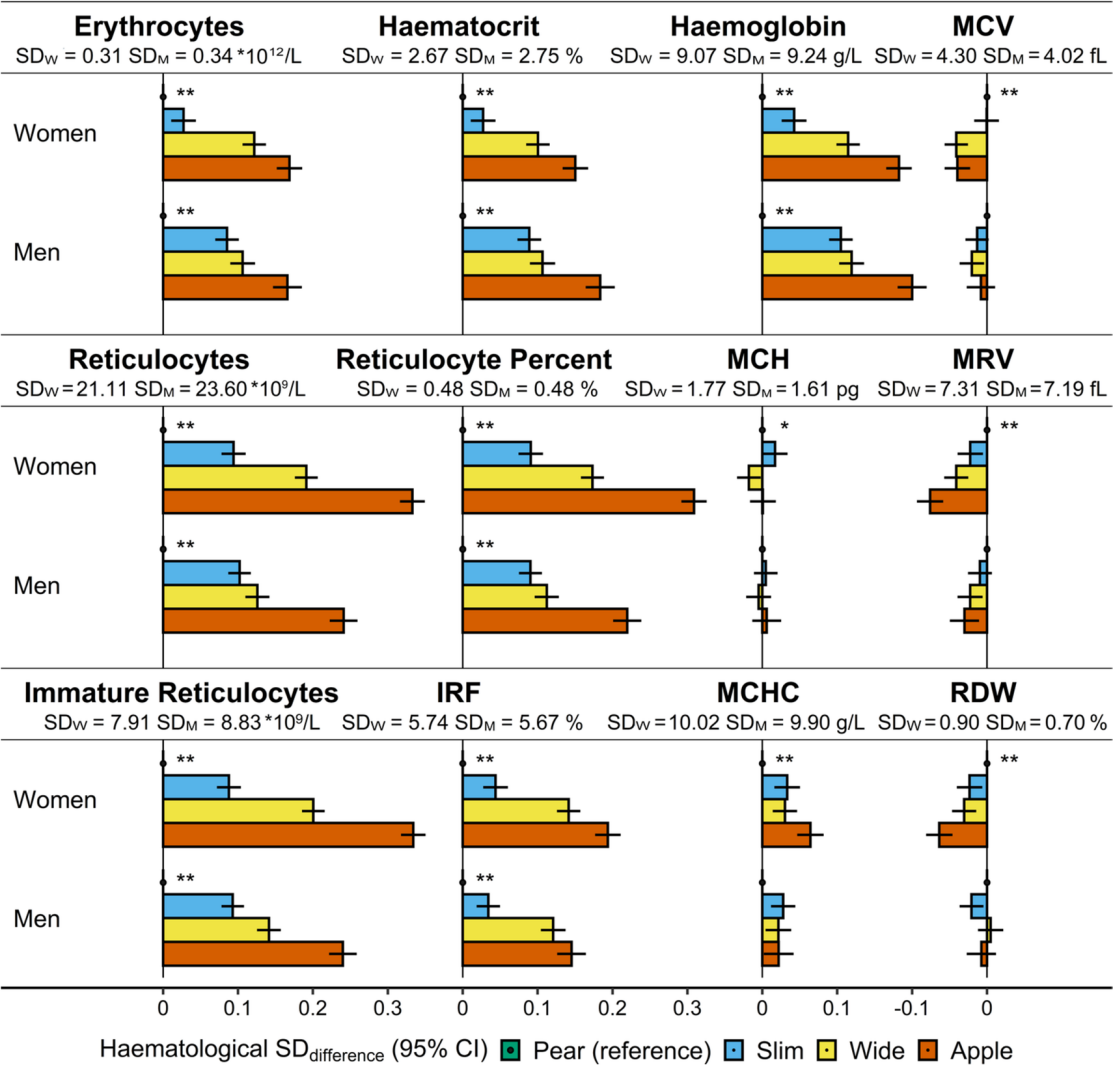
Associations of body shape phenotypes with erythrocyte and reticulocyte parameters. ABSI – a body shape index (cut-offs: ≥ 73 women, ≥ 80 men); Apple – large-ABSI-small-HI; BMI – body mass index; CI – confidence interval; HI – hip index (cut-offs: ≥ 64 women, ≥ 49 men); IRF – immature reticulocyte fraction; MCH – mean corpuscular haemoglobin; MCHC – mean corpuscular haemoglobin concentration; MCV – mean corpuscular volume; MRV – mean reticulocyte volume; Pear – small-ABSI-large-HI; RDW – red cell distribution width; SD – standard deviation (SD_W_ – women; SD_M_ – men); Slim – small-ABSI-small-HI; Wide – large-ABSI-large-HI. SD_difference_ (95% CI) – regression coefficient (95% confidence interval) interpreted for each body shape category as the difference in a haematological parameter on SD scale compared to the reference category ("pear”). Estimates were obtained from multivariable linear regression models, including each erythrocyte or reticulocyte parameter as an outcome variable (sex-specific z-scores, value minus mean divided by SD). Exposure variables included an ABSI-by-HI cross-classification (“pear” reference) and BMI categories. Adjustment variables included height, age, recent weight change, smoking status, alcohol consumption, physical activity, Townsend deprivation index, dietary intake (fruit, vegetables, red meat, processed meat, poultry, fish, cheese, bread, cereals, tea, coffee), recent major dietary change, region of the assessment centre, time of blood collection, fasting time, use of nonsteroidal anti-inflammatory drugs, use of vitamins ACE, vitamin D and minerals, or multivitamins, and for women, menopausal status, hormonal replacement therapy use, oral contraceptives use, and age at the last live birth. Values are shown in Supplementary Table S4. p _next to “pear”_ – p-value for the association with body shape overall, from a likelihood ratio test (separately for women and men), comparing a fully adjusted model including BMI categories, with a model additionally including ABSI-by-HI. * *p* < 0.001; ** *p* < 1*10^–6^

Corresponding to the positive associations with ABSI and inverse with HI, erythrocyte count, haematocrit, haemoglobin, and reticulocyte counts and proportions were highest for “apple” and lowest for “pear” phenotype in both women and men (Fig. 2). Associations of body shape phenotypes with cellular haemoglobin content and size were weak and limited to women, with the lowest levels in “apple” phenotype for MRV and RDW and the highest levels in “apple” phenotype for MCHC, but similarly lower MCV in “wide” and “apple” compared to “pear” phenotype (Fig. 2).

**Fig. 3:**
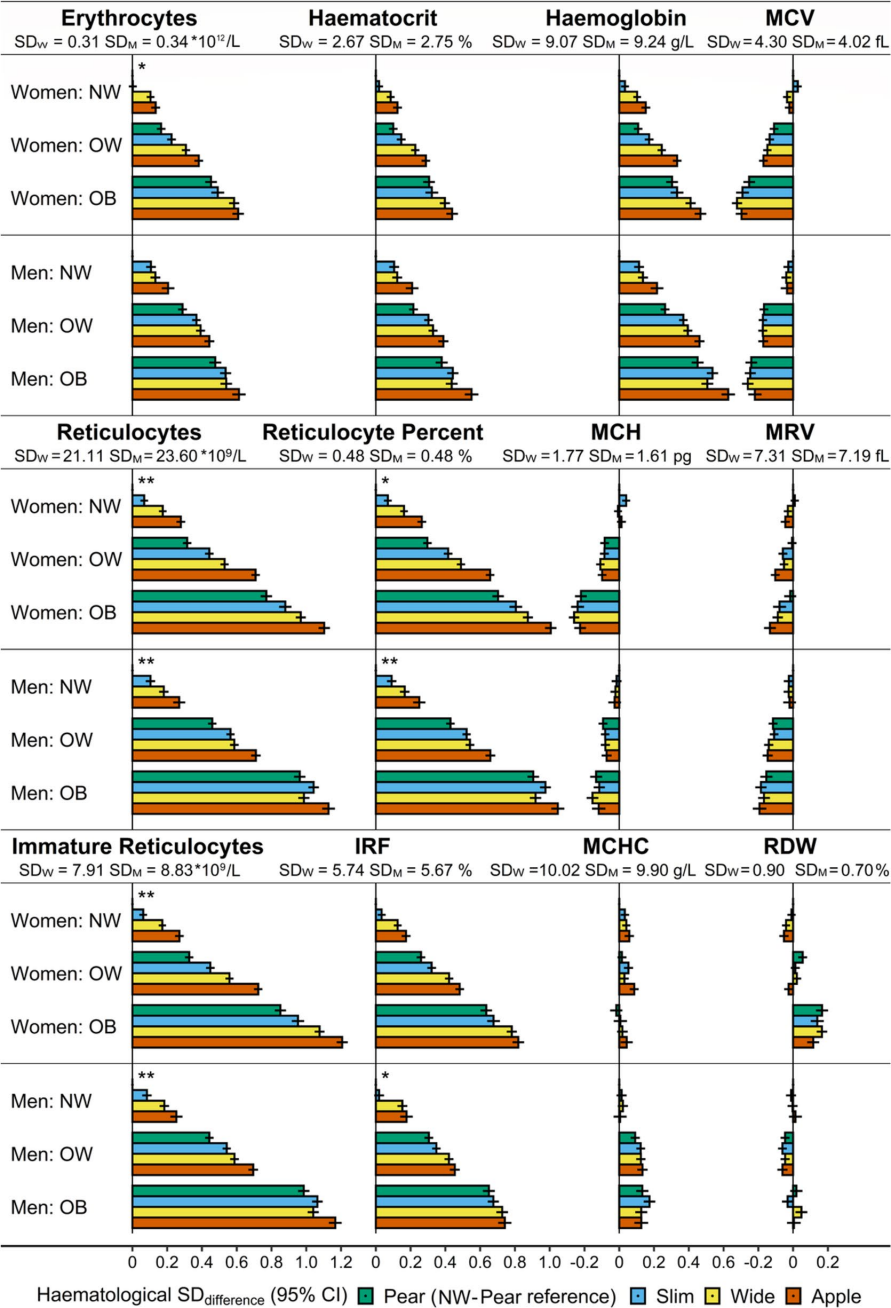
Heterogeneity according to BMI of associations of body shape phenotypes with erythrocyte and reticulocyte parameters. ABSI – a body shape index (cut-offs: ≥ 73 women, ≥ 80 men); Apple – large-ABSI-small-HI; BMI – body mass index; CI – confidence interval; HI – hip index (cut-offs: ≥ 64 women, ≥ 49 men); IRF – immature reticulocyte fraction; MCH – mean corpuscular haemoglobin; MCHC – mean corpuscular haemoglobin concentration; MCV – mean corpuscular volume; MRV – mean reticulocyte volume; NW – normal weight (BMI ≥ 18.5 to < 25 kg/m^2^); OW – overweight (BMI ≥ 25 to < 30 kg/m^2^); OB – obese (BMI ≥ 30 to < 45 kg/m^2^); Pear – small-ABSI-large-HI; RDW – red cell distribution width; SD – standard deviation (SD_W_ – women; SD_M_ – men); Slim – small-ABSI-small-HI; Wide – large-ABSI-large-HI. SD_difference_ (95% CI) – regression coefficient (95% confidence interval) interpreted for each anthropometric category as the difference in a haematological parameter on SD scale compared to the reference category (NW-"pear”). Estimates were obtained from multivariable linear regression models, including each erythrocyte or reticulocyte parameter as an outcome variable (sex-specific z-scores, value minus mean divided by SD). An BMI-by-ABSI-by-HI cross-classification represented the exposure variable (a single model for all BMI categories, reference NW-“pear”). Adjustment variables included height, age, recent weight change, smoking status, alcohol consumption, physical activity, Townsend deprivation index, dietary intake (fruit, vegetables, red meat, processed meat, poultry, fish, cheese, bread, cereals, tea, coffee), recent major dietary change, region of the assessment centre, time of blood collection, fasting time, use of nonsteroidal anti-inflammatory drugs, use of vitamins ACE, vitamin D and minerals, or multivitamins, and for women, menopausal status, hormonal replacement therapy use, oral contraceptives use, and age at the last live birth. Values are shown in Supplementary Table S4. p _next to NW-“pear”_ – *p*-value for heterogeneity of the association with body shape according to BMI category, from a likelihood ratio test (separately for women and men) comparing the fully adjusted additive model including ABSI-by-HI and BMI categories, with the interaction model including BMI-by-ABSI-by-HI. * *p* < 0.001; ** *p* < 1*10^–6^

Combining obesity and body shape phenotypes in fully adjusted models, haemoglobin was over 0.4 SD higher for obese-“apple” compared to normal-weight-“pear” phenotype (women: SD_difference_ = 0.47; 95% confidence interval 0.43–0.50; SD_W_ = 9.1 g/L; men: 0.63; 0.59–0.67; SD_M_ = 9.2 g/L), erythrocyte count was some 0.6 SD higher (women: 0.61; 0.57–0.64; SD_W_ = 0.31*10^12^/L; men: 0.61; 0.57–0.65; SD_M_ = 0.34*10^12^/L), total reticulocyte count was some 1.1 SD higher (women: 1.10; 1.07–1.14; SD_W_ = 21.1*10^9^/L; men: 1.13; 1.09–1.17; SD_M_ = 23.6*10^9^/L), immature reticulocyte count was some 1.2 SD higher (women: 1.21; 1.17–1.24; SD_W_ = 7.9*10^9^/L; men: 1.17; 1.13–1.20; SD_M_ = 8.8*10^9^/L), total reticulocyte percent was some 1.0 SD higher (women: 1.01; 0.97–1.04; SD_W_ = 0.48%; men: 1.05; 1.01–1.08; SD_M_ = 0.48%), and IFR was over 0.7 SD higher (women: 0.82; 0.79–0.85; SD_W_ = 5.74%; men: 0.74; 0.70–0.78; SD_M_ = 5.67%) (Fig. 3, Supplementary Table S4). Although levels were highest for “apple” and lowest for “pear” in each BMI category, the difference between “apple” and “pear” was largest for overweight in women but was gradually smaller for overweight and smallest for obese in men (Supplementary Table S4).

On an absolute scale, unadjusted IRF was some 20% higher in obese-“apple” compared to normal-weight-“pear” (mean = 31.3 (SD = 5.7) vs 26.5 (5.5) in women; 30.5 (5.5) vs 25.9 (5.5) in men; unit %), unadjusted reticulocyte count was some 50% higher (68.7 (22.9) vs 44.8 (17.7) in women; 76.6 (24.7) vs 48.6 (19.5) in men; unit *10^9^/L), as was unadjusted reticulocyte percent (1.55 (0.51) vs 1.06 (0.41) in women; 1.57 (0.50) vs 1.04 (0.41) in men; unit %), while unadjusted immature reticulocyte count was almost twice as high (21.9 (9.3) vs 12.1 (6.0) in women; 23.8 (10.0) vs 12.9 (6.5) in men; unit *10^9^/L), with only minimal differences for the remaining erythrocyte and reticulocyte parameters (Table 1).

**Table 1:** Anthropometric characteristics and erythrocyte and reticulocyte parameters of obesity and body shape phenotypes

	**NORMAL WEIGHT**		**OVERWEIGHT**		**OBESE**	
	**Pear**	**Apple**	**Pear**	**Apple**	**Pear**	**Apple**
**WOMEN**						
Cohort: n (% sex)	14,886 (14.1)	8357 (7.9)	9530 (9.0)	9724 (9.2)	4094 (3.9)	4217 (4.0)
Age (years)	54.2 (7.9)	56.0 (7.9)	55.4 (7.9)	56.8 (7.6)	54.8 (7.9)	55.6 (7.8)
BMI (kg/m^2^)	22.6 (1.6)	22.9 (1.5)	27.0 (1.4)	27.2 (1.4)	33.8 (3.4)	32.9 (2.6)
ABSI	69.7 (2.3)	76.7 (3.2)	69.6 (2.5)	77.1 (3.1)	69.7 (2.6)	77.1 (3.1)
HI	65.8 (1.3)	62.5 (1.3)	65.8 (1.4)	62.3 (1.4)	66.2 (1.7)	62.0 (1.7)
Erythrocytes 10^12^/L	4.24 (0.30)	4.28 (0.32)	4.30 (0.31)	4.37 (0.31)	4.39 (0.30)	4.44 (0.32)
Haematocrit %	38.7 (2.6)	39.2 (2.7)	39.0 (2.6)	39.6 (2.6)	39.5 (2.6)	40.0 (2.7)
Reticulocytes 10^9^/L	44.8 (17.7)	50.6 (19.7)	51.8 (19.5)	60.1 (21.4)	61.6 (21.8)	68.7 (22.9)
Reticulocyte Percent %	1.06 (0.41)	1.18 (0.45)	1.21 (0.45)	1.38 (0.49)	1.41 (0.49)	1.55 (0.51)
Imm. Reticulocytes 10^9^/L	12.1 (6.0)	14.3 (7.1)	14.8 (7.1)	18.0 (8.2)	19.1 (8.8)	21.9 (9.3)
IRF %	26.5 (5.5)	27.5 (5.5)	28.0 (5.5)	29.3 (5.6)	30.2 (5.7)	31.3 (5.7)
Haemoglobin g/L	133.0 (8.8)	134.9 (9.2)	134.2 (8.7)	136.6 (9.1)	135.8 (8.7)	137.6 (9.4)
MCH pg	31.4 (1.7)	31.6 (1.8)	31.3 (1.7)	31.3 (1.7)	31.0 (1.7)	31.1 (1.8)
MCHC g/L	344.0 (10.2)	344.7 (10.0)	344.2 (9.9)	345.0 (9.3)	343.8 (9.4)	344.5 (9.2)
MCV fL	91.3 (4.2)	91.6 (4.5)	90.9 (4.2)	90.9 (4.3)	90.2 (4.2)	90.1 (4.4)
MRV fL	105.5 (7.1)	105.5 (7.3)	105.5 (7.3)	105.0 (7.4)	105.3 (7.5)	104.6 (7.3)
RDW %	13.4 (0.9)	13.4 (0.9)	13.5 (0.9)	13.4 (0.9)	13.6 (0.9)	13.5 (0.9)
**MEN**						
Cohort: n (% sex)	9471 (9.4)	3790 (3.8)	13,589 (13.5)	8394 (8.3)	4620 (4.6)	3362 (3.3)
Age (years)	54.3 (8.3)	56.4 (7.8)	53.6 (8.1)	56.9 (7.7)	52.9 (7.8)	55.7 (7.7)
BMI (kg/m^2^)	23.1 (1.4)	23.3 (1.4)	27.2 (1.4)	27.3 (1.4)	32.9 (2.8)	32.6 (2.5)
ABSI	76.7 (2.5)	82.7 (2.3)	77.0 (2.3)	82.6 (2.1)	77.2 (2.2)	82.7 (2.2)
HI	50.2 (0.9)	48.0 (0.9)	50.1 (0.9)	48.0 (0.9)	50.2 (1.1)	47.9 (0.9)
Erythrocytes 10^12^/L	4.66 (0.34)	4.71 (0.35)	4.76 (0.33)	4.80 (0.34)	4.83 (0.33)	4.87 (0.34)
Haematocrit %	42.7 (2.7)	43.4 (2.9)	43.3 (2.7)	43.8 (2.7)	43.9 (2.6)	44.3 (2.7)
Reticulocytes 10^9^/L	48.6 (19.5)	54.9 (20.7)	60.2 (22.2)	65.7 (22.5)	73.1 (24.5)	76.6 (24.7)
Reticulocyte Percent %	1.04 (0.41)	1.17 (0.44)	1.26 (0.45)	1.37 (0.46)	1.51 (0.50)	1.57 (0.50)
Imm. Reticulocytes 10^9^/L	12.9 (6.5)	15.2 (7.4)	17.1 (8.0)	19.3 (8.5)	22.3 (9.7)	23.8 (10.0)
IRF %	25.9 (5.5)	27.1 (5.5)	27.8 (5.5)	28.8 (5.5)	30.0 (5.5)	30.5 (5.5)
Haemoglobin g/L	147.8 (9.0)	149.9 (9.6)	150.3 (8.8)	152.1 (9.1)	152.3 (8.8)	153.8 (9.2)
MCH pg	31.8 (1.5)	31.9 (1.7)	31.6 (1.5)	31.8 (1.6)	31.6 (1.6)	31.7 (1.6)
MCHC g/L	346.1 (9.6)	346.0 (9.3)	347.1 (10.0)	347.2 (9.6)	347.5 (9.8)	347.2 (9.1)
MCV fL	91.8 (3.9)	92.2 (4.4)	91.2 (3.8)	91.5 (4.1)	90.8 (4.0)	91.2 (4.0)
MRV fL	106.4 (7.1)	106.9 (7.5)	105.6 (7.1)	105.9 (7.3)	105.3 (7.1)	105.4 (7.3)
RDW %	13.3 (0.7)	13.4 (0.7)	13.3 (0.7)	13.3 (0.7)	13.3 (0.7)	13.4 (0.7)

*ABSI* a body shape index (cut-offs ≥ 73 for women, ≥ 80 for men); *Apple* large-ABSI-small-HI, *BMI* body mass index, *HI* hip index (cut-offs ≥ 64 for women, ≥ 49 for men), *Imm. Reticulocytes* immature reticulocytes, *IRF* immature reticulocyte fraction, *MCH* mean corpuscular haemoglobin, *MCHC* mean corpuscular haemoglobin concentration, *MCV* mean corpuscular volume, *MRV* mean reticulocyte volume; Normal weight – BMI ≥ 18.5 to < 25 kg/m^2^; Overweight – BMI ≥ 25 to < 30 kg/m^2^; Obese – BMI ≥ 30 to < 45 kg/m^2^; Pear – small-ABSI-large-HI; RDW – red cell distribution width. Values represent mean (standard deviation). Pairwise comparisons for obese-“apple” vs. normal-weight-“pear” were performed with unpaired t-test. All comparisons were significant at *p* < 1*10^–6^, except for MCHC in women (*p* = 0.01) and MCH (*p* = 1*10^–4^) and RDW (*p* = 0.002) in men

### Sensitivity analyses

Association estimates from fully adjusted models were higher for higher BMI categories and for higher tertiles of ABSI and HI, compatible with linearity (Supplementary Figure S2). The only suggestions for non-linearity were similar positive associations of BMI with MCHC for overweight and obese compared to normal weight men and a stronger positive association with RDW for obese than for overweight compared to normal weight women. Examining individually BMI, ABSI, and HI influenced little association estimates (see model “Individual index” in Supplementary Figure S3). Omitting all covariates also had little influence on association estimates, except for stronger positive associations of ABSI with haematocrit and haemoglobin in women, a weaker positive association of ABSI with erythrocyte count in men, and additional positive associations of ABSI with MCH, MCV, MRV, and RDW in men (see model “Additive (Unadjusted)” in Supplementary Figure S3). In the unrestricted dataset, all positive associations with BMI were retained and were only weaker for erythrocyte count, haemoglobin, and haematocrit, and in men also for reticulocyte counts, but were stronger for RDW even in men. Associations with ABSI and HI were largely retained in the unrestricted dataset (see model “General UK Biobank population” in Supplementary Figure S3).

## Discussion

In our cross-sectional study, uncomplicated obesity was associated with increased erythropoiesis, as indicated by high haemoglobin, haematocrit, erythrocyte and reticulocyte counts, and with reticulocyte immaturity, as indicated by high IRF. There were little differences between women and men, or between pre-menopausal and post-menopausal women.

Our study confirms, in a large healthy middle-aged cohort, higher haemoglobin levels for higher BMI, in agreement with a systematic review including women and men [8] and subsequent individual smaller-scale studies [9, 22–25]. Our findings differ from small-scale studies reporting lower haemoglobin for obesity in pre-menopausal women or when combining women and men [26, 27]. Our study also confirms positive associations of erythrocyte count and haematocrit with BMI, previously reported only in isolated small-scale studies [24, 25]. Our findings are further in agreement with a previous whole-blood gene-expression analysis reporting a shift from mature erythrocytes towards reticulocytes and upregulation of genes involved in haem synthesis for higher BMI [28].

A key factor stimulating erythropoiesis is erythropoietin (EPO) [29]. Literature reports relating EPO to obesity, however, are mainly focused on exogenous EPO administration, which reduces fat accumulation and improves glycaemic characteristics [30], and little information is available about endogenous EPO in obesity. Notably, however, in chronic kidney disease, when EPO production is restricted due to kidney damage, haemoglobin levels are higher for higher BMI [31] and lower EPO doses are required to maintain haemoglobin levels in obese compared to normal weight patients [32–34]. This suggests that a factor alternative to EPO is involved in the stimulation of erythropoiesis in obesity, with a prominent candidate being leptin, which is produced by adipose tissue [35]. In agreement, obesity induced with high-fat diet in mice leads to higher bone marrow adipocyte infiltration and higher leptin levels, with a similar increase of red and white cell precursors [36]. Furthermore, leptin levels are lower in patients with end-stage kidney disease requiring higher EPO doses to maintain haemoglobin levels compared to patients managing to maintain haemoglobin with lower EPO doses [37]. Glucocorticoids may also contribute to stimulated erythropoiesis, as they are associated with both general and abdominal obesity [38] and are important regulators of erythropoiesis [39]. Glucocorticoid receptor activation stimulates erythroid progenitor self-renewal but inhibits terminal differentiation [40–42], which would be compatible with the shift towards immature cells that we have described. Furthermore, oestrogens, which are derived in obesity via peripheral aromatisation in subcutaneous adipose tissue [43], increase iron availability and facilitate erythropoiesis by suppressing hepcidin production [44, 45], and increase haematopoietic stem-cell renewal [46]. Nevertheless, oestrogens inhibit hypoxia induced EPO production in animal models [47] and circulating oestradiol was higher in men and post-menopausal women with large HI [48], which is compatible with the inverse associations of erythrocyte and reticulocyte counts with hip size described in our study.

Our study is the largest cross-sectional study to date in healthy individuals without known underlying comorbidities or use of exogenous hormones and medications. This enabled us to investigate separately women and men, subgroups according to menopausal status, and complex two-way and three-way cross-classifications according to anthropometric indices. Although exclusion of participants with comorbidities and obesity complications does introduce selection bias with respect to the general UK Biobank population, we aimed to examine associations with uncomplicated obesity and not with comorbidities. We have also shown that the positive associations were largely retained in the general UK Biobank population, with only a small attenuation. This may reflect the fact that UK Biobank participants have a healthier lifestyle and potentially fewer obesity-related complications and comorbidities compared to the general UK population [49]. We were able to examine non-overlapping anthropometric indices reflecting obesity and body shape in joint models and to examine body shape within individual BMI categories without introducing collider bias because ABSI and HI are complementary to BMI, as BMI is factored out of waist and hip circumferences to derive, correspondingly, ABSI and HI [11, 12]. A further strength of our study is the adjustment for major lifestyle, reproductive and dietary factors, and use of supplements, which minimised confounding. Anthropometric measurements were obtained according to standardised procedures, thus avoiding bias from self-reporting. Blood samples were collected, stored, and processed according to standardised protocols and haematological parameters were measured with a single type of automatic blood count analyser, thus minimising measurement errors or differences. We also had information on reticulocyte parameters.

Our study, however, is subject to the key limitation of observational and especially of cross-sectional studies, that the described associations may not be causal. There was no follow-up dataset of matching size to assess temporality and, therefore, our findings would need to be validated in prospective studies examining changes in erythrocyte and reticulocyte parameters in association with changes in BMI and body shape, as well as in intervention studies examining changes in erythropoiesis in response to obesity modifying strategies. Also, regrettably, there was no information on iron or oxygenation status, nor an assessment of erythrocyte lifespan, nor measurements of adipokines, erythropoietin, or glucocorticoids. Participants younger than 40 and older than 70 years were not available either. Underweight and severely obese participants were few, as were participants with different from white background and could not be investigated adequately. Disease status and medication use were self-reported and may be incomplete or misclassified, potentially affecting the exclusions. Last, we could not examine associations with body composition, as only 1% of participants had erythrocyte and reticulocyte parameters assessed at the imaging visit, during which body composition measurements were obtained with dual-energy X-ray absorptiometry (DXA) or magnetic resonance imaging (MRI).

## Conclusion

General and abdominal obesity uncomplicated with diabetes are associated with increased erythropoiesis and reticulocyte immaturity.

## Supplementary Information

**Additional file 1.** 

## Data Availability

The dataset analysed in the current study was used under license and cannot be made freely available in a public repository or obtained from the authors due to restrictions related to privacy regulations and informed consent of the participants. Access to the data, however, can be obtained by *bona fide* researchers from UK Biobank, subject to approval of the research project and a material transfer agreement. For information on how to apply for gaining access to UK Biobank data, please follow the instructions at https://www.ukbiobank.ac.uk/enable-your-research For further queries related to the data contact the corresponding author Dr Sofia Christakoudi s.christakoudi@imperial.ac.uk.

## Abbreviations

ABSI: A body shape index
BMI: Body mass index
CI: Confidence interval
HC: Hip circumference
HI: Hip index
HR: Hazard ratio
MCV: Mean corpuscular volume
MCH: Mean corpuscular haemoglobin
MCHC: Mean corpuscular haemoglobin concentration
MRV: Mean reticulocyte volume
NHANES: National Health and Nutrition Examination Survey
RDW: Red cell distribution width
SD: Standard deviation
WC: Waist circumference
