# Supplementary material for: Associations of obesity and body shape with erythrocyte and reticulocyte parameters in the UK Biobank cohort
Source: BMC Endocr Disord. 2023 Aug 2;23:161. doi: 10.1186/s12902-023-01423-1 (PMC10394790; doi:10.1186/s12902-023-01423-1)
Supplement: Supplementary file 1 — Additional file 1. [file 12902_2023_1423_MOESM1_ESM.pdf]

Associations of obesity and body shape with erythrocyte and reticulocyte parameters in the UK Biobank cohort

Sofia Christakoudi, Konstantinos K. Tsilidis, Evangelos Evangelou, Elio Riboli

Supplementary Methods

Definition of variables.. 2

Statistical tests for heterogeneity.. 8

Supplementary Tables

Supplementary Table S1 Flow chart of UK Biobank participants in the study 9

Supplementary Table S2 Characteristics of study participants by sex and menopausal status.... 12

Supplementary Table S3 Associations of obesity and body shape indices with erythrocyte and reticulocyte parameters 14

Supplementary Table S4 Associations of obesity and body shape phenotypes with erythrocyte and reticulocyte parameters 16

Supplementary Figures

Supplementary Figure S1 Associations of obesity and body shape indices with erythrocyte and reticulocyte parameters according to menopausal status.......... 21

Supplementary Figure S2 Associations of obesity and body shape index categories with erythrocyte and reticulocyte parameters 23

Supplementary Figure S3A Associations of obesity and body shape indices with erythrocyte and reticulocyte parameters: sensitivity analyses (women).............. 25

Supplementary Figure S3B Associations of obesity and body shape indices with erythrocyte and reticulocyte parameters: sensitivity analyses (men) 26

References.. 28

Supplementary Methods

Definition of variables

The following variables were defined according to our previous study on associations of body shape and body size with cancer development [ref. 16]: prevalent and incident cancers and deaths (used for exclusions), age at recruitment, region of the assessment region, weight change during the last year preceding enrolment, smoking status, alcohol consumption, physical activity, Townsend deprivation index, and in women, hormone replacement therapy (HRT) use, and age at the last live birth (used for adjustment).

The following variables were defined according to our previous study on associations of body shape and body size with metabolic and inflammatory biomarkers [ref. 13]: fasting time, time of blood collection, and in women, menopausal status, and oral contraceptives use (used for adjustment), as well as diabetes and use of lipid-lowering and anti-hypertensive medications (used for exclusions).

The variables below were defined specifically for this study. The questions associated with each field can be found at the UK Biobank showcase:

https://biobank.ndph.ox.ac.uk/showcase/Iron or erythropoietin (for exclusions) was based on Fields [6179-0.0…5] "*Mineral and other dietary supplements*", coding values as Yes for Answer: 5 "*Iron*", as No for answers 1,2,3,4,6, or -7, and considering missing the remaining. Further information was obtained from Fields [20084-0.0…20] "*Vitamin and/or mineral supplement use*", coding Answer: 469 "*Multivitamin with iron*" OR Answer: 486 "*Iron*" as Yes. In addition, values were coded as Yes based on Fields [20003-0.0…47] "*Treatment/medication code*" with the following codes (UK Biobank Coding 4):

1140858274	ferromyn 100mg tablet	1140870410	ferrous gluc+folic acid+ascorbic acid 250mg/5mg/10mg tablet
1140858276	ironorm 116mg/ml drops	1140870412	polysaccharide iron complex
1140858290	ironedetate na 27.5mg/5ml elixir	1140870474	sodium ironedetate
1140858294	bc-500 with iron tablet	1140870476	sytron 190mg/5ml elixir
1140858298	feospan-z m/r capsule	1140870480	fefol-vit spansule
1140858300	ferrograd-c filmtab	1140870482	fefol z spansule
1140858302	ferromyn-s tablet	1140870484	ferrocap 330mg m/r capsule
1140858304	fesovit m/r capsule	1140870498	jectofer injection
1140858306	fesovit-z m/r capsule	1140870568	erythropoietin product
1140858376	irofol-c m/r tablet	1140870600	epoetin alfa
1140858378	ironorm capsule	1140870604	eprex 4000iu/1ml injection
1140870294	feospan 150mg spansule	1140870618	epoetin beta
1140870296	ferrograd 325mg filmtab	1140870626	recormon 1000iu injection+diluent
1140870298	slow-fe 160mg m/r tablet	1140876608	feroglobin b12 syrup
1140870306	iron+folic acid	1140888386	iron product
1140870308	fefol spansule	1140888390	ferrous salt product
1140870310	ferfolic sv tablet	1140888912	ferrocontin continus 454mg m/r tablet
1140870312	ferrocap-f 350 m/r capsule	1140910436	feso4 - ferrous sulphate

1140870314	ferrocontin folic continus tablet	1140910548	iron sulphate
1140870316	ferrograd folic filmtab	1140921160	fersaday 304mg tablet
1140870320	galfer fa capsule	1141157448	epoetin alfa product
1140870324	lexpec with iron syrup	1141171788	ferriprox 500mg tablet
1140870330	slow-fe folic m/r tablet	1141177686	fersamal tablet
1140870354	fersaday 322mg tablet	1141178816	aranesp 10micrograms/0.4ml prefilled syringe
1140870376	fergon 300mg tablet	1141178858	darbepoetin alfa
1140870382	plesmet syrup	1141182094	feredetate na 27.5mg/5ml elixir
1140870390	ferrous sulphate		

Vitamin B12 or folic acid (for exclusions) was based on Fields [6155-0.0…5] “*Vitamin and mineral supplements*”, coding values as Yes for Answer: 6 “*Folic acid or Folate (Vit B9)*”, as No for answers 1,2,3,4,5,7, or -7, and considering missing the remaining. Further information was obtained from Fields [20084-0.0…20] “*Vitamin and/or mineral supplement use*”, coding Answer: 477 “*Vitamin B12*” OR Answer 481: “*Folic acid*” as Yes. In addition, values were coded as Yes based on Fields [20003-0.0…47] “*Treatment/medication code*” with the following codes (UK Biobank Coding 4):

1140858282	fe-cap folic capsule	1140870486	folicin tablet
1140858452	hepacon b12 1mg/1ml injection	1140870504	hydroxocobalamin product
1140870318	folex-350 tablet	1140870508	cobalin-h 1mg/1ml injection
1140870326	meterfolic tablet	1140870512	cyanocobalamin product
1140870328	pregaday tablet	1140870516	cytacon 50micrograms tablet
1140870422	folic acid product	1140870520	cytamen 1mg/1ml injection
1140870428	lexpec 2.5mg/5ml sugar free syrup	1140870570	vitamin b12 preparation
1140870432	preconceive 400micrograms tablet	1140910494	b12 - hydroxocobalamin prep
1140870436	folinic acid product	1140910778	cantassium folic acid 400micrograms tablet
1140870438	calcium leucovorin 15mg tablet	1140912228	b12 - cyanocobalamin prep
1140870446	refolinon 15mg tablet	1141168788	folicare 400micrograms/5ml s/f oral solution

Vitamins ACE (for adjustment) was based on Fields [6155-0.0…5] “*Vitamin and mineral supplements*”, coding values as Yes for Answer: 1 “*Vitamin A*”, OR 3 “*Vitamin C*”, OR 5 “*Vitamin E*”, as No for answers 2,4,6,7, or -7, and considering missing the remaining. Further information was obtained from Fields [20084-0.0…20] “*Vitamin and/or mineral supplement use*”, coding as Yes Answer: 475 “*Vitamin A*” OR 478 “*Vitamin C*” OR 480 “*Vitamin E*”. In addition, values were coded as Yes based on Fields [20003-0.0…47] “*Treatment/medication code*” with the following codes (UK Biobank Coding 4):

1185	vitamin e product [ctsu]	1140871028	becosym tablet
1195	vitamin c product	1140871030	becosym forte tablet
1140852756	vitamin a	1140871112	vitamin e product
1140870852	vitamin a product	1140876590	alpha tocopheryl succinate
1140870932	ascorbic acid product	1140888346	alpha tocopheryl product
1140870946	redoxon 25mg tablet	1140909726	vitamin c product
1140871004	vitamins b+c	1140910780	cantassium vitamin e 200iu capsule

Vitamin D was based on Fields [6155-0.0…5] "*Vitamin and mineral supplements*", coding values as Yes for Answer: 4 "*Vitamin D*", as No for answers 1,2,3,5,6,7, or -7, and considering missing the remaining. Further information was obtained from Fields [20084-0.0…20] "*Vitamin and/or mineral supplement use*", coding as Yes Answer: 479 "*Vitamin D*". In addition, values were coded as Yes based on Fields [20003-0.0…47] "*Treatment/medication code*" with the following codes (UK Biobank Coding 4):

1140852766	vitamin a+d capsule	1140877630	calcium+ergocalciferol tablet
1140852946	calciferol high-str. 10,000units tablet	1140877632	calciferol 250micrograms tablet
1140852948	calcium+vitamin d 500units tablet	1140877696	abidec drops
1140870954	vitamin d product	1140880086	calcipotriol
1140870956	alfacalcidol	1140909592	calcijex 1microgram/1ml injection
1140870958	one-alpha 250nanograms capsule	1140909876	calciferol
1140870976	alfa-d 250nanograms capsule	1140909878	vitamin d2
1140870980	calcitriol product	1140909880	hydroxycholecalciferol
1140870982	rocaltrol 250nanograms capsule	1140909882	dihydroxycholecalciferol
1140871046	calciferol 7.5mg(300,000units)/1ml injection	1140923728	tacalcitol
1140871052	calcium carbonate+cholecalciferol 1.25g/5micrograms tablet	1141146606	calcium carbonate+cholecalciferol 1.25g/200iu tablet
1140871054	dihydrotachysterol	1141146612	calceos chewable tablet
1140871056	at 10 250micrograms/ml oral solution	1141172148	ergocalciferol 7.5mg(300,000units)/1ml injection
1140876588	ergocalciferol product	1141174388	calcitriol
1140877612	vitamin c+a+d tablet	1141180936	calcium carbonate+colecalciferol 1.25g/5micrograms tablet

Minerals was based on Fields [6179-0.0…5] "*Mineral and other dietary supplements*", coding values as Yes for Answer: 3 "*Calcium*", OR 4 "*Zinc*" OR 6 "*Selenium*", as No for answers 1,2,5, or -7, and considering missing the remaining. Further information was obtained from Fields [20084-0.0…20] "*Vitamin and/or mineral supplement use*", coding as Yes Answer: 470 "*Multivitamin with calcium*", OR 482 "*Chromium*", OR 483 "*Magnesium*", OR 484 "*Selenium*", OR 485 "*Calcium*" OR 487 "*Zinc*". In addition, values were coded as Yes based on Fields [20003-0.0…47] "*Treatment/medication code*" with the following codes (UK Biobank Coding 4):

1140852836	cacit 500mg effervescent tablet	1140870862	calcichew forte 2.5g chewable tablet
1140852884	calcimax syrup	1140870886	sodium cellulose phosphate
1140857200	calcium sulphaloxate	1140870888	calcisorb 5g/sachet powder
1140870574	calcium resonium powder	1140870900	phosphate-sandoz tablet
1140870576	resonium a powder	1140871050	calcichew d3 tablet
1140870788	calcium salts	1140876646	calcium polystyrene sulphonate
1140870796	calcium-sandoz syrup	1140883748	selenium product
1140870798	ossopan 830mg tablet	1140888538	zinc product
1140870800	sandocal 400 effervescent tablet	1140909530	zincosol 220mg effervescent tablet
1140870840	solvazinc 200mg effervescent tablet	1140911682	selenium ace tablet
1140870842	zincomed 220mg capsule	1140923682	tricalcium phosphate 3.3g/sachet powder
1140870844	z span 61.8mg spansule	1140923684	ostram 1.2g/sachet powder
1140870856	calcichew 1.25g chewable tablet	1141157472	calcium polystyrene sulphonate product
1140870860	calcidrink 2.52g/sachet granules		

Vitamin D or minerals (for adjustment) was defined as Yes when either vitamin D or minerals were "*Yes*" and No otherwise.

Multivitamins (for adjustment) was based on Fields [6155-0.0…5] "*Vitamin and mineral supplements*", coding values as Yes for Answer: 7 "*Multivitamins +/- minerals*", as No for answers 1,2,3,4,5,6, or -7, and considering missing the remaining. Further information was obtained from Fields [20084-0.0…20] "*Vitamin and/or mineral supplement use*", coding as Yes Answer: 468 "*Multivitamin*" OR 471 "*Multivitamin with multimineral*". In addition, values were coded as Yes based on Fields [20003-0.0…47] "*Treatment/medication code*" with the following codes (UK Biobank Coding 4):

1140852908	multivite pellet	1140871162	vitamins capsule bpc
1140852976	multivitamins	1140876592	multivitamin+mineral preparations
1140870488	forceval capsule	1140909766	multivitamins capsule
1140870492	octovit tablet	1141164602	mothers' and children's vitamin drops

Nonsteroidal anti-inflammatory drugs (NSAID) (for adjustment) was based, as in [ref. 10], on Fields [6154-0.1/5] "*Medication for pain relief, constipation, heartburn*", coding values as Yes for Answer 1: "*Aspirin*" OR Answer 2: "*Ibuprofen*", as No for Answers: 3,4,5,6, or -7, and considering missing the remaining. In addition, values were coded as Yes based on Fields [20003-0.0…47] "*Treatment/medication code*" with the following codes (UK Biobank Coding 4, including medication codes in Supplementary Table S1 of [ref. 13], but extended with medications additional to Aspirin and Ibuprofen).

1140856412	norgesic tablet	1140871582	pirozip 10 capsule
1140861766	ethamsylate	1140871590	flamatrol 10mg capsule
1140861804	angettes 75mg tablet	1140871604	sulindac
1140861806	aspirin 75mg tablet	1140871606	clinoril 100mg tablet
1140861808	disprin cv 100mg m/r tablet	1140871614	tiaprofenic acid
1140861832	tranexamic acid	1140871616	surgam 200mg tablet
1140861834	cyklokapron 500mg tablet	1140871628	prosaid 250mg tablet
1140864860	nu-seals aspirin 75mg e/c tablet	1140871638	napratec tablet combination pack
1140865578	mesalazine	1140871654	phenylbutazone product
1140865668	sulphasalazine	1140871660	butacote 100mg e/c tablet
1140865670	salazopyrin 500mg tablet	1140871662	butazone 100mg tablet
1140868226	aspirin	1140871666	piroxicam
1140868258	aspav dispersible tablet	1140871672	feldene 10mg capsule
1140868282	aspirin+methocarbamol 325mg/400mg tablet	1140872040	aspirin+metoclopramide 325mg/5mg effervescent tablet
1140868336	synflex 275mg tablet	1140875336	nabumetone
1140871080	benorylate	1140875338	relifex 500mg tablet
1140871082	benoral 750mg tablet	1140875346	tenoxicam
1140871092	salsalate	1140875546	piroxicam 0.5% gel
1140871094	disalcid 500mg capsule	1140875630	movelat cream
1140871100	azapropazone	1140875632	movelat gel
1140871102	rheumox 300mg capsule	1140875640	traxam gel
1140871168	voltarol 25mg e/c tablet	1140875642	ketoprofen 2.5% gel
1140871174	voltarol 100mg suppository	1140877880	fepron 600mg tablet
1140871180	rhumalgan 25mg e/c tablet	1140878030	ibuprofen+codeine phosphate
1140871188	etodolac	1140878036	diclofenac sodium+misoprostol
1140871202	fenbufen	1140879502	olsalazine
1140871206	lederfen 300mg tablet	1140881612	naproxen+misoprostol

1140871218	fenbuzip 300mg tablet
1140871226	fenoprofen
1140871228	fenopron 300mg tablet
1140871236	flurbiprofen
1140871238	froben 50mg tablet
1140871248	volraman 25mg e/c tablet
1140871256	valenac 25mg e/c tablet
1140871260	diclozip-25 e/c tablet
1140871266	arthrotec tablet
1140871274	isclofen 50mg e/c tablet
1140871276	flamrase 25mg e/c tablet
1140871282	diflunisal
1140871284	dolobid 250mg tablet
1140871310	ibuprofen
1140871320	arthrofen 200 tablet
1140871336	indomethacin
1140871344	artracin 25mg capsule
1140871348	imbrilon 25mg capsule
1140871354	indocid 25mg capsule
1140871360	flexin-25 continus m/r tablet
1140871370	apsifen 200mg tablet
1140871374	brufen 200mg tablet
1140871386	ebufac 200mg tablet
1140871388	cuprofen 200mg tablet
1140871392	isisfen 400mg tablet
1140871394	fenbid 300mg spansule
1140871396	lidifen 200mg tablet
1140871402	codafen continus m/r tablet
1140871404	junifen 100mg/5ml s/f suspension
1140871406	motrin 200mg tablet
1140871408	ibumed 400mg tablet
1140871416	rimafen 200mg tablet
1140871430	rimacid 25mg capsule
1140871434	indomod 25mg m/r capsule
1140871442	indomax 25 capsule
1140871454	contraflam 250mg capsule
1140871462	naproxen
1140871468	laraflex 250mg tablet
1140871472	naprosyn 250mg tablet
1140871482	rheuflex-250 tablet
1140871484	valrox 250mg tablet
1140871490	pranoxen continus 375mg m/r tablet
1140871506	ketoprofen
1140871516	orudis 50mg capsule
1140871522	oruvail 100 m/r capsule
1140871528	ketovail 100mg m/r capsule
1140871532	ketonal 50mg capsule
1140871542	mefenamic acid
1140871546	ponstan 250mg capsule
1140871556	arthroxen 250mg tablet
1140871564	rimoxyn 250mg tablet
1140871568	nycopren 250mg e/c tablet

1140882108	aspirin+cyclizine hydrochloride 500mg/25mg tablet
1140882190	aspirin+glycine 500mg/133mg dispersible tablet
1140882268	aspirin+codeine 300mg/8mg tablet
1140882392	aspirin+codeine
1140883812	parfenac 5% cream
1140884488	diclofenac
1140884498	felbinac
1140909702	sulfasalazine
1140909772	acetylsalicylic acid
1140909776	etamsylate
1140910496	propionic acid-ibuprofen
1140910598	5asa - mesalazine
1140910600	aminosalicylic acid
1140910678	azodisalicylic acid
1140910680	disodium azodisalicylic acid
1140910686	hydroxyphenylbutazone
1140911748	ibuprofen+menthol 5%/3% gel
1140911750	deep relief ibuprofen gel
1140911754	anadin tablet
1140911756	askit powder
1140925806	aceclofenac
1140925808	preservex 100mg tablet
1140926732	meloxicam
1141145722	slofenac sr 75mg m/r tablet
1141149110	cuprofen 5% gel
1141153134	anadin ibuprofen 200mg tablet
1141157412	ibuprofen product
1141157452	indomethacin product
1141163138	aspirin+papaveretum 500mg/7.71mg dispersible tablet
1141164044	isosorbide mononitrate+aspirin
1141164746	dexketoprofen
1141164750	keral 25mg tablet
1141165574	galprofen 100mg/5ml oral suspension
1141165754	librofem 200mg tablet
1141167844	dipyridamole+aspirin
1141167848	asasantin retard m/r capsule
1141169526	piroxicam-beta-cyclodextrin
1141169530	brexidol 20mg tablet
1141182674	fenactol 25mg e/c tablet
1141182708	ipocol 400mg e/c tablet
1141182754	piroxicam-betadex
1141184156	lornoxicam
1141184162	xefo 4mg tablet
1141184290	vioxxacute 25mg tablet
1141184292	vioxxacute 50mg tablet
1141184546	ibuprofen+pseudoephedrine hydrochloride
1141187776	nurofen 200mg tablet
1141190952	cuprofen plus tablet
1141191742	calprofen 100mg/5ml s/f oral suspension
1141194296	lemsip flu 12hr ibuprofen+pseudoephedrine capsule
1141200748	care ibuprofen 10% gel

Fruit intake (for adjustment) was based on the sum of Field [1309-0.0] "*Fresh fruit intake*" and Field [1319-0.0] "*Dried fruit intake*" .

Vegetable intake (for adjustment) was based on the sum of Field [1289-0.0] "*Cooked vegetable intake*" and Field [1299-0.0] *"Salad / raw vegetable intake"*.

Red meat intake (for adjustment) was based on the sum of Field [1369-0.0] "*Beef intake*", Field [1379-0.0] "*Lamb/mutton intake*", and Field [1389-0.0] "*Pork intake*"..

Processed meat intake (for adjustment) was based on Field [1349-0.0] "*Processed meat intake*".

Poultry intake (for adjustment) was based on Field [1359-0.0] "*Poultry intake*".

Fish intake (for adjustment) was based on the sum of Field [1329-0.0] "*Oily fish intake*" and Field [1339-0.0] "*Non-oily fish intake*"

For all meat variables above, the categorical answers were converted to a continuous scale as follows: Answer 0 "*Never*" remained 0; Answer 1 "*Less than once a week*" was coded as 0.5; Answer 2 "*Once a week*" was coded as 1; Answer 3 "*2-4 times a week*" was coded as 3; Answer 4 "*5-6 times a week*" was coded as 5.5; Answer 5 "*Once or more daily*" was coded as 7.

Cheese intake (for adjustment) was based on Field [1408-0.0] "*Cheese intake*". The categorical answers were converted to a continuous scale as for meat variables. Additional information was obtained from Field [6144-0.0] "*Never eat eggs, dairy, wheat, sugar*". Participants providing Answer 2: "*Dairy products*" were recoded as 0.

Bread intake (for adjustment) was based on field: Field [1438-0.0] "*Bread intake*".

Cereal intake (for adjustment) was based on field: Field [1458-0.0] "*Cereal intake*".

Tea intake (for adjustment) was based on Field [1488-0.0] "*Tea intake*".

Coffee intake (for adjustment) was based on Field [1498-0.0] "*Coffee intake*".

For all dietary variables, Answer: -10 *"Less than one"* was re-coded to 0.5 and Answers: -1 "*Do not know*" and -3 "*Prefer not to answer*" were considered missing.

Diet change (for exclusions and adjustment) was based on Field [1538-0.0] "*Major dietary changes in the last 5 years*". Participants providing Answers: 1 "*Yes, because of illness*"; -3 "*prefer not to answer*", and those with missing values were excluded from the study. Answer 2: "*Yes, because of other reason*" was coded as Yes. Answer: 0 "*No*" was coded as No.

Statistical tests for heterogeneity

ABSI – a body shape index; **BMI** – body mass index; **HI** – hip index.

To evaluate associations with body shape phenotypes overall, we used a likelihood ratio test comparing a model including BMI categories and covariates with a model additionally including ABSI-by-HI cross-classification, separately in women and men.

To evaluate heterogeneity of the associations with body shape phenotypes according to BMI, we used a likelihood ratio test comparing the additive model, including body shape phenotypes (ABSI-by-HI cross-classification), BMI categories, and covariates, with the interaction model, including BMI-by-ABSI-by-HI cross-classification and covariates, separately in women and men.

To evaluate heterogeneity in the associations with BMI according to sex, we used the interaction term between BMI (sex-specific z-scores) and sex (women reference) from a joint model, including women and men, adjusted for ABSI and HI (sex-specific z-scores) and all covariates (except female-specific). To evaluate heterogeneity in the associations with body shape indices according to sex, we used the interaction terms between ABSI and sex and HI and sex from a joint model, including women and men, adjusted for BMI and all covariates (except female-specific). To account for potential differences by menopausal status in women, we additionally included an interaction term between age and sex in all models examining differences according to sex.

To evaluate heterogeneity in the associations with BMI according to menopausal status, we used the interaction term between BMI (sex-specific z-scores) and menopausal status (pre-menopausal reference) from a joint model, including pre-menopausal and post-menopausal women, adjusted for ABSI and HI (sex-specific z-scores) and all covariates (including female-specific). To evaluate heterogeneity in the associations with body shape indices according to menopausal status, we used the interaction terms between ABSI and menopausal status and HI and menopausal status from a joint model, including pre-menopausal and post-menopausal women, adjusted for BMI and all covariates (including female-specific).

Supplementary Table S1 Flow chart of UK Biobank participants in the study

	Exclusions for the main analysis dataset	Women	Men
	**Total:**	**273,328**	**229,084**
1.	Ethnic background (restricted to self-reported white) [a]	15,936	13,861
2.	Anthropometric measurements missing or extreme [b]	4758	2372
3.	Age restrictions (age <40 or >70 years)	2	9
4.	Genetic & self-reported sex mismatch or sex chromosome aneuploidy [a]	386	396
5.	Pregnant or unknown [a]	293	0
6.	Prevalent cancer at enrolment [a]	21,844	12,182
7.	Cancer or death within two years post enrolment [a]	3464	4078
8.	Diabetes (self-reported) or anti-diabetic medication at enrolment [c]	6828	12,349
9.	Glycated haemoglobin HbA1c≥48 mmol/mol	912	1563
10.	Thyroid-related illness [d]	19,122	3222
11.	Pituitary, adrenal, other endocrine illness [d]	431	224
12.	Liver or kidney related illness [d]	1802	1986
13.	Inflammatory bowel disease [d]	1695	1428
14.	Systemic or immunological disorder [e]	4242	2269
15.	C-reactive protein CRP≥10 mg/L	6326	4989
16.	Chronic respiratory illness or heart failure [d]	4090	4773
17.	Haematological disorders [f]	2858	1065
18.	Lipid-lowering medications [g]	16,640	29,757
19.	Anti-hypertensive medications [g]	15,720	14,129
20.	Current HRT or oral contraceptives use at enrolment [h]	15,488	0
21.	Glucocorticoids (oral) [g]	465	473
22.	Iron or erythropoietin containing medications [i]	6592	4026
23.	Vitamin B12 or folic acid containing medications [i]	2456	1324
24.	Detected nucleated red blood cells	1160	795
25.	Blood sample >28 days post enrolment	1	0
26.	Missing any erythrocyte or reticulocyte measurements	6903	5430
27.	Extreme erythrocyte or reticulocyte parameters [j]	305	222
27.	Major dietary changes in the last 5 years due to illness	4845	3394
28.	Missing recent weight or diet change information	1911	1914
	**Total excluded for the main dataset:**	**167,475**	**128,230**
	%	(61.3)	(56.0)

	Exclusions for the unrestricted dataset	Women	Men
1.	Anthropometric measurements missing	1538	1709
2.	Erythrocyte of reticulocyte measurements missing	18,572	12,886
	**Total excluded for the unrestricted dataset:**	**20,110**	**14,595**
	%	(7.4)	(6.4)

WOMEN	MEN
**Main analysis dataset: 105,853**	**Main analysis dataset: 100,854**
Unrestricted dataset: 253,218	Unrestricted dataset: 214,489

Pre-MP	Post-MP
31,457 (29.7)	64,285 (60.7)

number (percent from sex); **HRT** – hormone replacement therapy.

The exclusion criteria were applied sequentially in the displayed order, counting each excluded individual only once.

[a] – for UK Biobank Field names, definition of variables, and definition of prevalent and incident cancer cases see Supplementary Methods in [ref. 16].

[b] – missing "a body shape index" (ABSI) or hip index (HI); height <130 cm; waist circumference <50 or >160 cm; body mass index (BMI) <18.5 or ≥45 kg/m^2. Field names for waist and hip circumferences, weight, and height are listed in Supplementary Methods of [ref. 16].

[c] – the ascertainment of prevalent diabetes status was based on a combination of self-reported illness and medication use (see Supplementary Methods of [ref. 13]).

[d] – self-reported non-cancer illnesses were based on Fields [20002-0.0…33] "*Non-cancer illness code, self-reported*" (codes correspond to UK Biobank Coding 6). For lists of codes and disorders per category, see the legend of Supplementary Figure S1 in [ref. 13]. Liver and kidney illness additionally included codes:

1519 kidney nephropathy
1520 iga nephropathy
1607 diabetic nephropathy
1608 nephritis
1609 glomerulnephritis

[e] – self-reported systemic and immunological disorders from Fields [20002-0.0…33] with codes:

1371 sarcoidosis
1372 vasculitis
1373 connective tissue disorder
1376 giant cell/temporal arteritis
1377 polymyalgia rheumatica
1378 wegners granulmatosis
1380 polyartertis nodosa
1381 systemic lupus erythematosis/sle
1383 dermatopolymyositis
1384 scleroderma/systemic sclerosis
1464 rheumatoid arthritis
1477 psoriatic arthropathy
1480 dermatomyositis
1481 polymyositis

[f] – self-reported haematological disorders from Fields [20002-0.0…33] with codes:

1327 low platelets/platelet disorder
1328 haemophilia
1330 iron deficiency anaemia
1331 pernicious anaemia
1332 aplastic anaemia
1339 sickle cell disease
1340 thalassaemia
1438 polycythaemia vera
1445 clotting disorder/excessive bleeding
1446 anaemia
1447 pancytopenia
1448 neutropenia/lymphopenia
1449 myeloproliferative disorder

1450	monoclonal gammopathy/not myeloma
1451	hereditary/genetic haematological disorder
1546	essential thrombocytosis
1658	myelofibrosis

[g] – self-reported medication use was based on Fields [20003-0.0…47] "*Treatment/medication code*" (UK Biobank Coding 4) for men and women, Fields [6177-0.0…2] "*Medication for cholesterol, blood pressure or diabetes*" for men, and Fields [6153-0.0…3] "*Medication for cholesterol, blood pressure, diabetes, or take exogenous hormones*" for women. See Supplementary Table S1 in [ref. 13] for list of medications per category. Lipid-lowering drugs additionally included codes (Coding 4):

1140865576	cholestyramine
1140909780	colestyramine
1141157416	cholestyramine product

[h] – based on a combination of fields, as described in Supplementary Methods of [ref. 16] for HRT use and [ref. 13] for oral contraceptives use.

[i] – see lists of medications in Supplementary Methods (above).

[j] – reticulocyte count >200*10^9/L; immature reticulocyte count >100*10^9/L; reticulocyte percent >5%; red cell distribution width >20%.

Supplementary Table S2 Characteristics of study participants by sex and menopausal status

	WOMEN	Pre-MP	Post-MP	p $_{MP}$	MEN	p $_{sex}$
Cohort: n (% per sex)	105,853 (51.2)	31,457 (29.7)	64,285 (60.7)		100,854 (48.8)	
**Anthropometry**: mean (SD)						
BMI: kg/m^2	26.1 (4.3)	25.8 (4.4)	26.1 (4.1)	$4*10^{-29}$	27.1 (3.7)	$<1*10^{-293}$
Weight: kg	69.4 (12.0)	69.8 (12.6)	69.0 (11.5)	$2*10^{-22}$	84.5 (12.8)	$<1*10^{-293}$
Height: cm	163.1 (6.2)	164.4 (6.2)	162.4 (6.1)	$<1*10^{-293}$	176.6 (6.7)	$<1*10^{-293}$
Waist circumference: cm	82.1 (10.8)	80.8 (10.9)	82.5 (10.6)	$2*10^{-121}$	94.6 (10.0)	$<1*10^{-293}$
Hip circumference: cm	101.9 (8.8)	101.6 (9.0)	101.9 (8.7)	$3*10^{-6}$	102.6 (6.7)	$2*10^{-90}$
ABSI	73.3 (4.8)	72.4 (4.6)	73.7 (4.8)	$<1*10^{-293}$	79.2 (4.0)	$<1*10^{-293}$
HI	64.3 (2.4)	64.1 (2.3)	64.4 (2.4)	$1*10^{-58}$	49.1 (1.6)	$<1*10^{-293}$
**BMI categories**: n (%)						
Normal weight	48,950 (46.2)	15,998 (50.9)	28,905 (45.0)	$3*10^{-76}$	30,220 (30.0)	$<1*10^{-293}$
Overweight	39,270 (37.1)	10,402 (33.1)	24,979 (38.9)		51,744 (51.3)	
Obese	17,633 (16.7)	5057 (16.1)	10,401 (16.2)		18,890 (18.7)	
**Body shape**: n (%)						
Pear	28,510 (26.9)	9509 (30.2)	16,264 (25.3)	$3*10^{-293}$	27,680 (27.4)	$<1*10^{-293}$
Slim	24,488 (23.1)	8803 (28.0)	13,365 (20.8)		31,201 (30.9)	
Wide	30,557 (28.9)	7197 (22.9)	20,512 (31.9)		26,427 (26.2)	
Apple	22,298 (21.1)	5948 (18.9)	14,144 (22.0)		15,546 (15.4)	
**Medication use**: n (%)						
NSAIDs	25,272 (23.9)	8924 (28.4)	13,642 (21.2)	$3*10^{-132}$	20,310 (20.1)	$3*10^{-93}$
Vitamins ACE	9906 (9.4)	2416 (7.7)	6567 (10.2)	$2*10^{-36}$	8825 (8.8)	$2*10^{-6}$
Vitamin D or minerals	14,030 (13.3)	2224 (7.1)	10,613 (16.5)	$<1*10^{-293}$	5774 (5.7)	$<1*10^{-293}$
Multivitamins	23,809 (22.5)	6532 (20.8)	14,873 (23.1)	$1*10^{-16}$	17,677 (17.5)	$1*10^{-174}$
**Diet change**: n (%)						
Yes	34,525 (32.6)	9907 (31.5)	20,881 (32.5)	0.002	27,086 (26.9)	$5*10^{-180}$
**Haematology**: mean (SD)						
Erythrocytes 10^{12}/L	4.31 (0.31)	4.25 (0.31)	4.34 (0.31)	$<1*10^{-293}$	4.76 (0.34)	$<1*10^{-293}$
Haematocrit %	39.2 (2.7)	38.5 (2.8)	39.5 (2.5)	$<1*10^{-293}$	43.5 (2.7)	$<1*10^{-293}$
Reticulocytes 10^9/L	53.3 (21.1)	53.1 (21.7)	53.0 (20.7)	0.808	61.3 (23.6)	$<1*10^{-293}$
Reticulocyte Percent	1.23 (0.48)	1.25 (0.50)	1.22 (0.47)	$3*10^{-15}$	1.28 (0.48)	$1*10^{-123}$
Imm. Reticulocytes 10^9/L	15.5 (7.9)	15.5 (8.2)	15.4 (7.7)	0.061	17.7 (8.8)	$<1*10^{-293}$
IRF %	28.2 (5.7)	28.3 (5.9)	28.2 (5.7)	0.088	28.1 (5.7)	$2*10^{-11}$
Haemoglobin g/L	134.9 (9.1)	132.3 (10.0)	136.0 (8.4)	$<1*10^{-293}$	150.8 (9.2)	$<1*10^{-293}$
MCH pg	31.3 (1.8)	31.2 (2.1)	31.4 (1.6)	$5*10^{-56}$	31.7 (1.6)	$<1*10^{-293}$
MCHC pg/L	344.4 (10.0)	344.1 (10.1)	344.5 (9.9)	$3*10^{-10}$	347.0 (9.9)	$<1*10^{-293}$
MCV fL	91.0 (4.3)	90.7 (5.0)	91.2 (3.9)	$3*10^{-58}$	91.4 (4.0)	$4*10^{-111}$
MRV fL	105.4 (7.3)	105.1 (7.6)	105.5 (7.2)	$5*10^{-19}$	105.9 (7.2)	$2*10^{-72}$
RDW %	13.4 (0.9)	13.5 (1.1)	13.4 (0.8)	$6*10^{-97}$	13.3 (0.7)	$3*10^{-206}$

ABSI – a body shape index; **BMI** – body mass index; **HI** – hip index; **IRF** – immature reticulocyte fraction; **MCH** – mean corpuscular haemoglobin; **MCHC** – mean corpuscular haemoglobin concentration; **MCV** – mean corpuscular volume; **MRV** – mean reticulocyte volume; **n (%)** – number (percent from total per column); **RDW** – red cell distribution width; **SD** – standard deviation.

Pairwise comparisons for men vs women (**p_{sex}**) and post-menopausal vs pre-menopausal women (**p_{MP}**) were performed with unpaired t-test for continuous variables and χ^2-test for categorical variables.

The lifestyle and reproductive characteristics of a similarly restricted UK Biobank dataset have previously been summarised by sex and menopausal status in Supplementary Table S2 of [ref.21]

Supplementary Table S3 Associations of obesity and body shape indices with erythrocyte and reticulocyte parameters

Sex	Index	Count	SD_{change} (95% CI)	SD_{change} (95% CI)	SD_{change} (95% CI)	SD_{change} (95% CI)
			**Erythrocytes**<br>SD_W = 0.31 SD_M = 0.34 $*10^{12}$/L	**Haematocrit**<br>SD_W = 2.67 SD_M = 2.75 %	**Haemoglobin**<br>SD_W = 9.07 SD_M = 9.24 g/L	**MCV**<br>SD_W = 4.30 SD_M = 4.02 fL
**Women**	BMI	105,853	0.188 (0.182 to 0.194)**	0.120 (0.114 to 0.126)**	0.120 (0.113 to 0.126)**	-0.116 (-0.122 to -0.110)**
**Men**	BMI	100,854	0.163 (0.157 to 0.169)**	0.128 (0.122 to 0.134)**	0.154 (0.147 to 0.160)**	-0.085 (-0.091 to -0.078)**
$p_{sex\ BMI}$			0.0002	0.0003	**$6*10^{-22}$**	**$2*10^{-10}$**
**Women**	ABSI	105,853	0.080 (0.074 to 0.086)**	0.069 (0.063 to 0.075)**	0.078 (0.072 to 0.084)**	-0.024 (-0.030 to -0.018)**
**Men**	ABSI	100,854	0.072 (0.066 to 0.079)**	0.076 (0.070 to 0.083)**	0.084 (0.077 to 0.090)**	-0.008 (-0.014 to -0.001)
$p_{sex\ ABSI}$			0.013	0.096	0.101	$1*10^{-5}$
**Women**	HI	105,853	-0.025 (-0.031 to -0.019)**	-0.024 (-0.029 to -0.018)**	-0.034 (-0.040 to -0.028)**	0.004 (-0.002 to 0.010)
**Men**	HI	100,854	-0.058 (-0.064 to -0.052)**	-0.064 (-0.070 to -0.057)**	-0.074 (-0.080 to -0.067)**	0.004 (-0.002 to 0.010)
$p_{sex\ HI}$			**$2*10^{-9}$**	**$2*10^{-15}$**	**$3*10^{-15}$**	0.498
			**Reticulocytes**<br>SD_W = 21.11 SD_M = 23.60 $*10^{9}$/L	**Reticulocyte Percent**<br>SD_W = 0.48 SD_M = 0.48 %	**MCH**<br>SD_W = 1.77 SD_M = 1.61 pg	**MRV**<br>SD_W = 7.31 SD_M = 7.19 fL
**Women**	BMI	105,853	0.324 (0.318 to 0.330)**	0.294 (0.288 to 0.300)**	-0.100 (-0.107 to -0.094)**	-0.026 (-0.032 to -0.019)**
**Men**	BMI	100,854	0.343 (0.337 to 0.349)**	0.322 (0.316 to 0.328)**	-0.047 (-0.053 to -0.040)**	-0.061 (-0.067 to -0.055)**
$p_{sex\ BMI}$			**$1*10^{-8}$**	**$2*10^{-13}$**	**$3*10^{-30}$**	**$2*10^{-13}$**
**Women**	ABSI	105,853	0.130 (0.124 to 0.135)**	0.118 (0.112 to 0.124)**	-0.010 (-0.016 to -0.004)*	-0.030 (-0.036 to -0.023)**
**Men**	ABSI	100,854	0.099 (0.092 to 0.105)**	0.090 (0.083 to 0.096)**	0.000 (-0.007 to 0.006)	-0.017 (-0.023 to -0.010)**
$p_{sex\ ABSI}$			**$1*10^{-10}$**	**$5*10^{-8}$**	0.0009	0.024
**Women**	HI	105,853	-0.077 (-0.083 to -0.071)**	-0.074 (-0.080 to -0.068)**	-0.008 (-0.014 to -0.002)	0.020 (0.014 to 0.026)**
**Men**	HI	100,854	-0.079 (-0.085 to -0.073)**	-0.071 (-0.077 to -0.065)**	-0.006 (-0.012 to 0.000)	0.012 (0.006 to 0.018)*
$p_{sex\ HI}$			0.818	0.580	0.767	0.106
			**Immature Reticulocytes**<br>SD_W = 7.91 SD_M = 8.83 $*10^{9}$/L	**IRF**<br>SD_W = 5.74 SD_M = 5.67 %	**MCHC**<br>SD_W = 10.02 SD_M = 9.90 g/L	**RDW**<br>SD_W = 0.90 SD_M = 0.70 %
**Women**	BMI	105,853	0.361 (0.355 to 0.367)**	0.261 (0.255 to 0.267)**	-0.006 (-0.013 to 0.000)	0.070 (0.063 to 0.076)**
**Men**	BMI	100,854	0.360 (0.354 to 0.366)**	0.239 (0.233 to 0.245)**	0.047 (0.041 to 0.054)**	0.006 (0.000 to 0.013)
$p_{sex\ BMI}$			0.139	0.0005	**$1*10^{-31}$**	**$1*10^{-36}$**
**Women**	ABSI	105,853	0.134 (0.129 to 0.140)**	0.088 (0.082 to 0.094)**	0.018 (0.012 to 0.024)**	-0.023 (-0.029 to -0.016)**
**Men**	ABSI	100,854	0.105 (0.099 to 0.111)**	0.083 (0.076 to 0.089)**	0.011 (0.005 to 0.018)	-0.001 (-0.008 to 0.006)
$p_{sex\ ABSI}$			**$2*10^{-10}$**	0.374	0.485	$6*10^{-6}$
**Women**	HI	105,853	-0.073 (-0.078 to -0.067)**	-0.034 (-0.040 to -0.028)**	-0.022 (-0.028 to -0.016)**	0.018 (0.012 to 0.024)**
**Men**	HI	100,854	-0.070 (-0.076 to -0.064)**	-0.026 (-0.032 to -0.020)**	-0.017 (-0.024 to -0.011)**	0.013 (0.007 to 0.019)*
$p_{sex\ HI}$			0.408	0.074	0.308	0.315

ABSI – a body shape index (SD_W=4.80, SD_M=4.01); **BMI** – body mass index (SD_W=4.27, SD_M=3.66 kg/m^2); **CI** – confidence interval; **Count** – number of participants per category; **HI** – hip index (SD_W=2.37, SD_M=1.61); **IRF** – immature reticulocyte fraction; **MCH** – mean corpuscular haemoglobin; **MCHC** – mean corpuscular haemoglobin concentration; **MCV** – mean corpuscular volume; **MRV** – mean reticulocyte volume; **MRV** – mean reticulocyte volume; **RDW** – red cell distribution width; **SD** – standard deviation (SD_W – women; SD_M – men).

SD_{change} (95% CI) – regression coefficient (95% confidence interval) interpreted as the change in haematological parameters on SD scale per one SD increase of each anthropometric index. Estimates were obtained from multivariable linear regression models including each erythrocyte or reticulocyte parameter as an outcome variable (sex-specific z-scores, value minus mean divided by SD). Exposure variables included BMI, ABSI, and HI (sex-specific z-scores). Adjustment variables included height, age, recent weight change, smoking status, alcohol consumption, physical activity, Townsend deprivation index, dietary intake (fruit, vegetables, red meat, processed meat, poultry, fish, cheese, bread, cereals, tea, coffee), recent major dietary change, region of the assessment centre, time of blood collection, fasting time, use of nonsteroidal anti-inflammatory drugs, use of vitamins ACE, vitamin D and minerals, or multivitamins, and for women, menopausal status, hormonal replacement therapy use, oral contraceptives use, and age at the last live birth.

Plots are shown in Figure 1.

$p_{sex\ BMI}$ – p-value for the interaction term between BMI and sex, from a joint model including women (reference) and men, with adjustment for ABSI, HI, and all covariates, except female-specific, but including an interaction term between age and sex, to account for potential differences by menopausal status in women ($p<1*10^{-6}$ bold).

$p_{sex\ ABSI\ /\ HI}$ – p-value for the interaction term between ABSI and sex or HI and sex, from a joint model including women (reference) and men, with adjustment for BMI and all covariates, except female-specific, but including an interaction term between age and sex.

***** $p<0.001$; ****** $p<1*10^{-6}$ from Wald test for the individual term.

Supplementary Table S4 Associations of obesity and body shape phenotypes with erythrocyte and reticulocyte parameters

Sex	Shape	Count	$SD_{difference}$ (95% CI)	$SD_{difference}$ (95% CI)	$SD_{difference}$ (95% CI)	$SD_{difference}$ (95% CI)
			**Erythrocytes** SD_W = 0.31 SD_M = 0.34 *10^{12}/L	**Haematocrit** SD_W = 2.67 SD_M = 2.75 %	**Haemoglobin** SD_W = 9.07 SD_M = 9.24 g/L	**MCV** SD_W = 4.30 SD_M = 4.02 fL
**Women** [A]	Pear	28,510	reference	reference	reference	reference
	Slim	24,488	0.027 (0.011 to 0.044)	0.027 (0.011 to 0.044)	0.042 (0.026 to 0.059)**	-0.001 (-0.017 to 0.016)
	Wide	30,557	0.122 (0.106 to 0.137)**	0.100 (0.085 to 0.116)**	0.115 (0.099 to 0.130)**	-0.041 (-0.057 to -0.025)**
	Apple	22,298	0.169 (0.152 to 0.186)**	0.150 (0.133 to 0.167)**	0.183 (0.165 to 0.200)**	-0.040 (-0.057 to -0.022)*
$p_{shape\ overall}$			**2*10^{-107}**	**3*10^{-80}**	**5*10^{-110}**	**2*10^{-9}**
**Women: NW** [B]	Pear	14,886	reference	reference	reference	reference
	Slim	12,167	0.002 (-0.021 to 0.025)	0.018 (-0.005 to 0.042)	0.033 (0.010 to 0.056)	0.027 (0.004 to 0.050)
	Wide	13,540	0.104 (0.082 to 0.127)**	0.085 (0.063 to 0.108)**	0.103 (0.081 to 0.125)**	-0.035 (-0.057 to -0.013)
	Apple	8,357	0.134 (0.108 to 0.159)**	0.125 (0.099 to 0.150)**	0.152 (0.127 to 0.178)**	-0.022 (-0.048 to 0.004)
**Women: OW** [B]	Pear	9,530	0.165 (0.140 to 0.190)**	0.100 (0.075 to 0.125)**	0.109 (0.084 to 0.134)**	-0.110 (-0.135 to -0.085)**
	Slim	9,046	0.225 (0.200 to 0.251)**	0.146 (0.121 to 0.172)**	0.172 (0.147 to 0.197)**	-0.135 (-0.160 to -0.109)**
	Wide	10,970	0.308 (0.284 to 0.332)**	0.227 (0.203 to 0.251)**	0.244 (0.220 to 0.269)**	-0.148 (-0.172 to -0.124)**
	Apple	9,724	0.382 (0.357 to 0.407)**	0.288 (0.263 to 0.313)**	0.333 (0.308 to 0.358)**	-0.171 (-0.196 to -0.146)**
**Women: OB** [B]	Pear	4,094	0.452 (0.418 to 0.486)**	0.306 (0.273 to 0.340)**	0.304 (0.270 to 0.338)**	-0.253 (-0.286 to -0.219)**
	Slim	3,275	0.491 (0.454 to 0.528)**	0.322 (0.285 to 0.359)**	0.332 (0.296 to 0.369)**	-0.291 (-0.328 to -0.254)**
	Wide	6,047	0.585 (0.555 to 0.614)**	0.397 (0.368 to 0.426)**	0.410 (0.381 to 0.440)**	-0.323 (-0.352 to -0.293)**
	Apple	4,217	0.608 (0.575 to 0.642)**	0.439 (0.406 to 0.472)**	0.467 (0.434 to 0.501)**	-0.296 (-0.330 to -0.263)**
$p_{BMI-by-shape}$			8*10^{-4}	0.046	0.018	0.008
**Men** [A]	Pear	27,680	reference	reference	reference	reference
	Slim	31,201	0.085 (0.070 to 0.101)**	0.089 (0.073 to 0.105)**	0.105 (0.089 to 0.121)**	-0.013 (-0.029 to 0.002)
	Wide	26,427	0.106 (0.090 to 0.123)**	0.107 (0.090 to 0.123)**	0.119 (0.102 to 0.136)**	-0.020 (-0.037 to -0.004)
	Apple	15,546	0.166 (0.147 to 0.185)**	0.184 (0.164 to 0.203)**	0.200 (0.181 to 0.219)**	-0.008 (-0.027 to 0.011)
$p_{shape\ overall}$			**2*10^{-69}**	**3*10^{-79}**	**1*10^{-96}**	0.094
**Men: NW** [B]	Pear	9,471	reference	reference	reference	reference
	Slim	8,507	0.107 (0.079 to 0.135)**	0.105 (0.077 to 0.134)**	0.114 (0.085 to 0.142)**	-0.028 (-0.055 to 0.000)
	Wide	8,452	0.132 (0.104 to 0.161)**	0.123 (0.095 to 0.152)**	0.136 (0.108 to 0.165)**	-0.039 (-0.067 to -0.011)
	Apple	3,790	0.205 (0.169 to 0.242)**	0.209 (0.172 to 0.246)**	0.217 (0.181 to 0.254)**	-0.035 (-0.071 to 0.001)
**Men: OW** [B]	Pear	13,589	0.288 (0.263 to 0.314)**	0.216 (0.191 to 0.242)**	0.263 (0.238 to 0.289)**	-0.168 (-0.193 to -0.142)**
	Slim	16,858	0.368 (0.344 to 0.393)**	0.303 (0.278 to 0.328)**	0.369 (0.344 to 0.393)**	-0.174 (-0.198 to -0.150)**
	Wide	12,903	0.392 (0.366 to 0.418)**	0.329 (0.303 to 0.355)**	0.395 (0.368 to 0.421)**	-0.174 (-0.200 to -0.149)**
	Apple	8,394	0.442 (0.414 to 0.471)**	0.388 (0.359 to 0.417)**	0.461 (0.432 to 0.490)**	-0.172 (-0.200 to -0.143)**
**Men: OB** [B]	Pear	4,620	0.477 (0.442 to 0.511)**	0.379 (0.344 to 0.413)**	0.451 (0.416 to 0.486)**	-0.240 (-0.274 to -0.207)**
	Slim	5,836	0.536 (0.504 to 0.568)**	0.443 (0.410 to 0.475)**	0.536 (0.504 to 0.569)**	-0.246 (-0.278 to -0.215)**
	Wide	5,072	0.540 (0.506 to 0.573)**	0.437 (0.403 to 0.471)**	0.507 (0.473 to 0.541)**	-0.261 (-0.294 to -0.228)**
	Apple	3,362	0.613 (0.574 to 0.651)**	0.551 (0.512 to 0.590)**	0.627 (0.588 to 0.666)**	-0.220 (-0.258 to -0.182)**
$p_{BMI-by-shape}$			0.065	0.093	0.025	0.353

Sex	Shape	Count	SDdifference (95% CI)	SDdifference (95% CI)	SDdifference (95% CI)	SDdifference (95% CI)
			**Reticulocytes** SDW = 21.11 SDM = 23.60 *10⁹/L	**Reticulocyte Percent** SDW = 0.48 SDM = 0.48 %	**MCH** SDW = 1.77 SDM = 1.61 pg	**MRV** SDW = 7.31 SDM = 7.19 fL
**Women** [A]	Pear	28,510	reference	reference	reference	reference
	Slim	24,488	0.094 (0.078 to 0.110)**	0.091 (0.075 to 0.107)**	0.017 (0.000 to 0.034)	-0.022 (-0.039 to -0.005)
	Wide	30,557	0.191 (0.176 to 0.206)**	0.173 (0.158 to 0.189)**	-0.018 (-0.034 to -0.002)	-0.041 (-0.057 to -0.025)**
	Apple	22,298	0.333 (0.316 to 0.349)**	0.309 (0.292 to 0.326)**	0.001 (-0.016 to 0.018)	-0.076 (-0.094 to -0.059)**
p shape overall			**<1*10^-298^**	**1*10^-298^**	7*10^-4^	**1*10^-16^**
**Women: NW** [B]	Pear	14,886	reference	reference	reference	reference
	Slim	12,167	0.068 (0.046 to 0.091)**	0.069 (0.046 to 0.092)**	0.040 (0.016 to 0.063)*	0.010 (-0.014 to 0.033)
	Wide	13,540	0.175 (0.153 to 0.196)**	0.162 (0.140 to 0.184)**	-0.007 (-0.030 to 0.015)	-0.030 (-0.053 to -0.007)
	Apple	8,357	0.279 (0.253 to 0.304)**	0.265 (0.239 to 0.290)**	0.013 (-0.013 to 0.039)	-0.045 (-0.071 to -0.018)*
**Women: OW** [B]	Pear	9,530	0.315 (0.291 to 0.339)**	0.297 (0.272 to 0.321)**	-0.084 (-0.109 to -0.059)**	-0.006 (-0.032 to 0.020)
	Slim	9,046	0.442 (0.418 to 0.467)**	0.416 (0.391 to 0.441)**	-0.085 (-0.111 to -0.060)**	-0.059 (-0.085 to -0.033)*
	Wide	10,970	0.530 (0.507 to 0.554)**	0.490 (0.466 to 0.513)**	-0.109 (-0.133 to -0.084)**	-0.052 (-0.077 to -0.027)*
	Apple	9,724	0.709 (0.684 to 0.733)**	0.657 (0.633 to 0.682)**	-0.098 (-0.124 to -0.073)**	-0.103 (-0.128 to -0.077)**
**Women: OB** [B]	Pear	4,094	0.771 (0.738 to 0.803)**	0.703 (0.670 to 0.737)**	-0.220 (-0.254 to -0.186)**	-0.017 (-0.051 to 0.018)
	Slim	3,275	0.879 (0.843 to 0.915)**	0.805 (0.769 to 0.842)**	-0.240 (-0.277 to -0.203)**	-0.078 (-0.116 to -0.040)*
	Wide	6,047	0.968 (0.940 to 0.997)**	0.875 (0.846 to 0.904)**	-0.260 (-0.290 to -0.230)**	-0.088 (-0.119 to -0.058)**
	Apple	4,217	1.103 (1.071 to 1.136)**	1.006 (0.973 to 1.039)**	-0.225 (-0.259 to -0.191)**	-0.133 (-0.168 to -0.099)**
p BMI-by-shape			**4*10^-7^**	9*10^-5^	0.169	0.003
**Men** [A]	Pear	27,680	reference	reference	reference	reference
	Slim	31,201	0.102 (0.087 to 0.117)**	0.090 (0.075 to 0.106)**	0.005 (-0.011 to 0.020)	-0.009 (-0.025 to 0.006)
	Wide	26,427	0.126 (0.110 to 0.142)**	0.112 (0.096 to 0.128)**	-0.005 (-0.022 to 0.012)	-0.023 (-0.039 to -0.006)
	Apple	15,546	0.241 (0.222 to 0.260)**	0.220 (0.201 to 0.238)**	0.006 (-0.013 to 0.025)	-0.030 (-0.050 to -0.011)
p shape overall			**1*10^-144^**	**4*10^-117^**	0.621	0.008
**Men: NW** [B]	Pear	9,471	reference	reference	reference	reference
	Slim	8,507	0.104 (0.077 to 0.131)**	0.091 (0.064 to 0.119)**	-0.015 (-0.044 to 0.013)	-0.025 (-0.053 to 0.004)
	Wide	8,452	0.180 (0.153 to 0.208)**	0.166 (0.139 to 0.194)**	-0.022 (-0.051 to 0.006)	-0.026 (-0.054 to 0.003)
	Apple	3,790	0.269 (0.234 to 0.304)**	0.250 (0.215 to 0.285)**	-0.027 (-0.063 to 0.010)	-0.020 (-0.057 to 0.017)
**Men: OW** [B]	Pear	13,589	0.459 (0.435 to 0.484)**	0.429 (0.404 to 0.454)**	-0.093 (-0.119 to -0.068)**	-0.116 (-0.142 to -0.090)**
	Slim	16,858	0.564 (0.541 to 0.588)**	0.522 (0.498 to 0.546)**	-0.081 (-0.105 to -0.056)**	-0.109 (-0.134 to -0.084)**
	Wide	12,903	0.586 (0.561 to 0.611)**	0.542 (0.516 to 0.567)**	-0.080 (-0.106 to -0.054)**	-0.140 (-0.167 to -0.114)**
	Apple	8,394	0.710 (0.683 to 0.738)**	0.659 (0.631 to 0.687)**	-0.073 (-0.102 to -0.044)**	-0.146 (-0.175 to -0.117)**
**Men: OB** [B]	Pear	4,620	0.962 (0.929 to 0.996)**	0.906 (0.872 to 0.939)**	-0.133 (-0.167 to -0.098)**	-0.154 (-0.189 to -0.119)**
	Slim	5,836	1.042 (1.011 to 1.073)**	0.975 (0.944 to 1.007)**	-0.115 (-0.147 to -0.083)**	-0.185 (-0.217 to -0.153)**
	Wide	5,072	0.986 (0.954 to 1.019)**	0.919 (0.886 to 0.951)**	-0.154 (-0.188 to -0.120)**	-0.167 (-0.201 to -0.133)**
	Apple	3,362	1.128 (1.091 to 1.165)**	1.047 (1.009 to 1.084)**	-0.118 (-0.157 to -0.079)**	-0.193 (-0.232 to -0.154)**
p BMI-by-shape			**1*10^-10^**	**3*10^-10^**	0.156	0.209

Sex	Shape	Count	$SD_{difference}$ (95% CI)	$SD_{difference}$ (95% CI)	$SD_{difference}$ (95% CI)	$SD_{difference}$ (95% CI)
			**Immature Reticulocytes**	**IRF**	**MCHC**	**RDW**
			$SD_W = 7.91$ $SD_M = 8.83$ $*10^9$/L	$SD_W = 5.74$ $SD_M = 5.67$ %	$SD_W = 10.02$ $SD_M = 9.90$ g/L	$SD_W = 0.90$ $SD_M = 0.70$ %
**Women** [A]	Pear	28,510	reference	reference	reference	reference
	Slim	24,488	0.088 (0.072 to 0.104)**	0.044 (0.027 to 0.060)**	0.033 (0.016 to 0.050)*	-0.023 (-0.040 to -0.006)
	Wide	30,557	0.201 (0.185 to 0.216)**	0.141 (0.126 to 0.157)**	0.030 (0.014 to 0.046)*	-0.030 (-0.047 to -0.014)*
	Apple	22,298	0.334 (0.318 to 0.350)**	0.194 (0.177 to 0.211)**	0.064 (0.047 to 0.082)**	-0.064 (-0.081 to -0.046)**
**p** shape overall			**$<1*10^{-298}$**	**$9*10^{-137}$**	**$5*10^{-11}$**	**$5*10^{-11}$**
**Women: NW** [B]	Pear	14,886	reference	reference	reference	reference
	Slim	12,167	0.062 (0.040 to 0.085)**	0.033 (0.010 to 0.056)	0.032 (0.008 to 0.056)	-0.009 (-0.033 to 0.015)
	Wide	13,540	0.172 (0.151 to 0.194)**	0.126 (0.103 to 0.148)**	0.040 (0.017 to 0.064)*	-0.041 (-0.064 to -0.018)*
	Apple	8,357	0.270 (0.245 to 0.295)**	0.174 (0.148 to 0.200)**	0.058 (0.031 to 0.084)*	-0.053 (-0.079 to -0.026)*
**Women: OW** [B]	Pear	9,530	0.327 (0.303 to 0.351)**	0.260 (0.235 to 0.285)**	0.016 (-0.010 to 0.041)	0.055 (0.029 to 0.081)*
	Slim	9,046	0.448 (0.424 to 0.473)**	0.321 (0.296 to 0.346)**	0.053 (0.027 to 0.079)*	0.013 (-0.014 to 0.039)
	Wide	10,970	0.558 (0.535 to 0.582)**	0.421 (0.397 to 0.446)**	0.030 (0.005 to 0.055)	0.021 (-0.004 to 0.046)
	Apple	9,724	0.724 (0.700 to 0.748)**	0.482 (0.458 to 0.507)**	0.086 (0.060 to 0.112)**	-0.026 (-0.051 to 0.000)
**Women: OB** [B]	Pear	4,094	0.851 (0.819 to 0.884)**	0.635 (0.602 to 0.669)**	-0.017 (-0.052 to 0.018)	0.166 (0.132 to 0.201)**
	Slim	3,275	0.952 (0.917 to 0.988)**	0.677 (0.640 to 0.714)**	0.007 (-0.032 to 0.045)	0.139 (0.101 to 0.177)**
	Wide	6,047	1.077 (1.048 to 1.105)**	0.782 (0.753 to 0.811)**	0.018 (-0.013 to 0.048)	0.166 (0.136 to 0.196)**
	Apple	4,217	1.207 (1.174 to 1.239)**	0.820 (0.786 to 0.853)**	0.043 (0.008 to 0.078)	0.116 (0.081 to 0.150)**
**p** BMI-by-shape			**$4*10^{-9}$**	0.228	0.482	0.086
**Men** [A]	Pear	27,680	reference	reference	reference	reference
	Slim	31,201	0.093 (0.078 to 0.108)**	0.034 (0.019 to 0.050)*	0.028 (0.012 to 0.044)*	-0.021 (-0.037 to -0.005)
	Wide	26,427	0.141 (0.125 to 0.157)**	0.121 (0.104 to 0.137)**	0.021 (0.004 to 0.039)	0.005 (-0.012 to 0.022)
	Apple	15,546	0.240 (0.222 to 0.258)**	0.145 (0.126 to 0.164)**	0.022 (0.002 to 0.042)	-0.008 (-0.027 to 0.012)
**p** shape overall			**$7*10^{-151}$**	**$2*10^{-71}$**	0.006	0.013
**Men: NW** [B]	Pear	9,471	reference	reference	reference	reference
	Slim	8,507	0.084 (0.057 to 0.111)**	0.019 (-0.009 to 0.047)	0.013 (-0.016 to 0.043)	-0.012 (-0.041 to 0.017)
	Wide	8,452	0.184 (0.156 to 0.211)**	0.153 (0.124 to 0.181)**	0.021 (-0.008 to 0.050)	-0.003 (-0.032 to 0.025)
	Apple	3,790	0.253 (0.218 to 0.288)**	0.176 (0.140 to 0.213)**	0.005 (-0.032 to 0.043)	0.013 (-0.024 to 0.050)
**Men: OW** [B]	Pear	13,589	0.442 (0.418 to 0.466)**	0.305 (0.280 to 0.330)**	0.092 (0.066 to 0.118)**	-0.046 (-0.072 to -0.020)*
	Slim	16,858	0.542 (0.519 to 0.566)**	0.348 (0.324 to 0.372)**	0.123 (0.098 to 0.149)**	-0.061 (-0.086 to -0.036)*
	Wide	12,903	0.587 (0.562 to 0.612)**	0.421 (0.395 to 0.447)**	0.124 (0.098 to 0.151)**	-0.046 (-0.072 to -0.019)*
	Apple	8,394	0.695 (0.667 to 0.722)**	0.454 (0.426 to 0.483)**	0.133 (0.104 to 0.163)**	-0.061 (-0.090 to -0.032)*
**Men: OB** [B]	Pear	4,620	0.985 (0.953 to 1.018)**	0.650 (0.616 to 0.684)**	0.133 (0.097 to 0.168)**	0.019 (-0.016 to 0.054)
	Slim	5,836	1.064 (1.033 to 1.095)**	0.676 (0.644 to 0.708)**	0.174 (0.141 to 0.207)**	-0.032 (-0.064 to 0.001)
	Wide	5,072	1.040 (1.008 to 1.072)**	0.727 (0.693 to 0.760)**	0.126 (0.092 to 0.161)**	0.048 (0.013 to 0.082)
	Apple	3,362	1.167 (1.130 to 1.204)**	0.742 (0.703 to 0.780)**	0.127 (0.087 to 0.167)**	0.005 (-0.035 to 0.044)
**p** BMI-by-shape			**$8*10^{-8}$**	$4*10^{-4}$	0.105	0.053

Sex	Shape	Count	$SD_{difference}$ (95% CI)	$SD_{difference}$ (95% CI)	$SD_{difference}$ (95% CI)	$SD_{difference}$ (95% CI)
			**Erythrocytes** SD_W = 0.31 SD_M = 0.34 $*10^{12}$/L	**Haematocrit** SD_W = 2.67 SD_M = 2.75 %	**Haemoglobin** SD_W = 9.07 SD_M = 9.24 g/L	**MCV** SD_W = 4.30 SD_M = 4.02 fL
**Women: NW** [B]	Apple vs Pear		0.134 (0.108 to 0.159)**	0.125 (0.099 to 0.150)**	0.152 (0.127 to 0.178)**	-0.022 (-0.048 to 0.004)
**OW** [B]	Apple vs Pear		0.216 (0.189 to 0.244)**	0.188 (0.161 to 0.215)**	0.224 (0.197 to 0.251)**	-0.061 (-0.088 to -0.034)*
**OB** [B]	Apple vs Pear		0.156 (0.115 to 0.197)**	0.133 (0.092 to 0.174)**	0.163 (0.122 to 0.205)**	-0.044 (-0.085 to -0.002)
**Men: NW** [B]	Apple vs Pear		0.205 (0.169 to 0.242)**	0.209 (0.172 to 0.246)**	0.217 (0.181 to 0.254)**	-0.035 (-0.071 to 0.001)
**OW** [B]	Apple vs Pear		0.154 (0.128 to 0.180)**	0.172 (0.146 to 0.199)**	0.198 (0.171 to 0.225)**	-0.004 (-0.030 to 0.022)
**OB** [B]	Apple vs Pear		0.136 (0.093 to 0.179)**	0.172 (0.129 to 0.216)**	0.176 (0.133 to 0.219)**	0.021 (-0.021 to 0.063)
			**Reticulocytes** SD_W = 21.11 SD_M = 23.60 $*10^{9}$/L	**Reticulocyte Percent** SD_W = 0.48 SD_M = 0.48 %	**MCH** SD_W = 1.77 SD_M = 1.61 pg	**MRV** SD_W = 7.31 SD_M = 7.19 fL
**Women: NW** [B]	Apple vs Pear		0.279 (0.253 to 0.304)**	0.265 (0.239 to 0.290)**	0.013 (-0.013 to 0.039)	-0.045 (-0.071 to -0.018)*
**OW** [B]	Apple vs Pear		0.394 (0.367 to 0.420)**	0.361 (0.334 to 0.387)**	-0.014 (-0.042 to 0.013)	-0.097 (-0.124 to -0.069)**
**OB** [B]	Apple vs Pear		0.333 (0.293 to 0.373)**	0.303 (0.262 to 0.344)**	-0.005 (-0.046 to 0.037)	-0.117 (-0.159 to -0.074)**
**Men: NW** [B]	Apple vs Pear		0.269 (0.234 to 0.304)**	0.250 (0.215 to 0.285)**	-0.027 (-0.063 to 0.010)	-0.020 (-0.057 to 0.017)
**OW** [B]	Apple vs Pear		0.251 (0.225 to 0.276)**	0.230 (0.204 to 0.256)**	0.021 (-0.006 to 0.047)	-0.030 (-0.057 to -0.004)
**OB** [B]	Apple vs Pear		0.166 (0.124 to 0.207)**	0.141 (0.099 to 0.183)**	0.015 (-0.028 to 0.058)	-0.039 (-0.083 to 0.004)
			**Immature Reticulocytes** SD_W = 7.91 SD_M = 8.83 $*10^{9}$/L	**Immature Ret. Fraction** SD_W = 5.74 SD_M = 5.67 %	**MCHC** SD_W = 10.02 SD_M = 9.90 g/L	**RDW** SD_W = 0.90 SD_M = 0.70 %
**Women: NW** [B]	Apple vs Pear		0.270 (0.245 to 0.295)**	0.174 (0.148 to 0.200)**	0.058 (0.031 to 0.084)*	-0.053 (-0.079 to -0.026)*
**OW** [B]	Apple vs Pear		0.397 (0.371 to 0.423)**	0.222 (0.195 to 0.249)**	0.071 (0.042 to 0.099)**	-0.081 (-0.109 to -0.053)**
**OB** [B]	Apple vs Pear		0.356 (0.316 to 0.395)**	0.184 (0.143 to 0.225)**	0.060 (0.017 to 0.103)	-0.051 (-0.093 to -0.008)
**Men: NW** [B]	Apple vs Pear		0.253 (0.218 to 0.288)**	0.176 (0.140 to 0.213)**	0.005 (-0.032 to 0.043)	0.013 (-0.024 to 0.050)
**OW** [B]	Apple vs Pear		0.253 (0.227 to 0.278)**	0.149 (0.123 to 0.176)**	0.042 (0.014 to 0.069)	-0.015 (-0.042 to 0.012)
**OB** [B]	Apple vs Pear		0.181 (0.140 to 0.222)**	0.091 (0.049 to 0.134)*	-0.006 (-0.050 to 0.039)	-0.014 (-0.058 to 0.029)

ABSI – a body shape index (cut-offs: ≥73 women, ≥80 men); **Apple** – large-ABSI-small-HI; **BMI** – body mass index; **CI** – confidence interval; **HI** – hip index (cut-offs: ≥64 women, ≥49 men); **IRF** – immature reticulocyte fraction; **MCH** – mean corpuscular haemoglobin; **MCHC** – mean corpuscular haemoglobin concentration; **MCV** – mean corpuscular volume; **MRV** – mean reticulocyte volume; **NW** – normal weight (BMI≥18.5 to <25 kg/m^2); **OW** – overweight (BMI≥25 to <30 kg/m^2); **OB** – obese (BMI≥30 to <45 kg/m^2); **Pear** – small-ABSI-large-HI; **RDW** – red cell distribution width; **SD** – standard deviation (SD_W – women; SD_M – men); **Slim** – small-ABSI-small-HI; **Wide** – large-ABSI-large-HI.

$SD_{difference}$ (95% CI) – regression coefficient (95% confidence interval) interpreted for each anthropometric category as the difference in a haematological parameter on SD scale compared to the reference category ("pear" for shape; NW-"pear" for BMI-by-shape). Estimates were obtained from multivariable linear regression models including each erythrocyte or reticulocyte parameter as an outcome variable (sex-specific z-scores, value minus mean divided by SD). Adjustment variables include height, age, recent weight change, smoking status, alcohol consumption, physical activity,

Townsend deprivation index, dietary intake (fruit, vegetables, red meat, processed meat, poultry, fish, cheese, bread, cereals, tea, coffee), recent major dietary change, region of the assessment centre, time of blood collection, fasting time, use of nonsteroidal anti-inflammatory drugs, use of vitamins ACE, vitamin D and minerals, or multivitamins, and for women, menopausal status, hormonal replacement therapy use, oral contraceptives use, and age at the last live birth. Plots are shown in Figure 2 (ABSI-by-HI) and Figure 3 (BMI-by-ABSI-by-HI).

A – a model including ABSI-by-HI cross-classification and BMI categories as exposure variables.

B – a model including BMI-by-ABSI-by-HI cross-classification as exposure variable.

$\mathbf{p}_{\textbf{shape overall}}$ – p-value for the association with body shape overall, from a likelihood ratio test (separately for women and men), comparing a fully adjusted model including BMI categories, with a model additionally including ABSI-by-HI.

$\mathbf{p}_{\textbf{BMI-by-shape}}$ – p-value for heterogeneity of the association with body shape according to BMI category, from a likelihood ratio test (separately for women and men), comparing the fully adjusted additive model including ABSI-by-HI and BMI categories, with the interaction model including BMI-by-ABSI-by-HI.

***** $p<0.001$; ****** $p<1*10^{-6}$ from Wald test for the individual term.

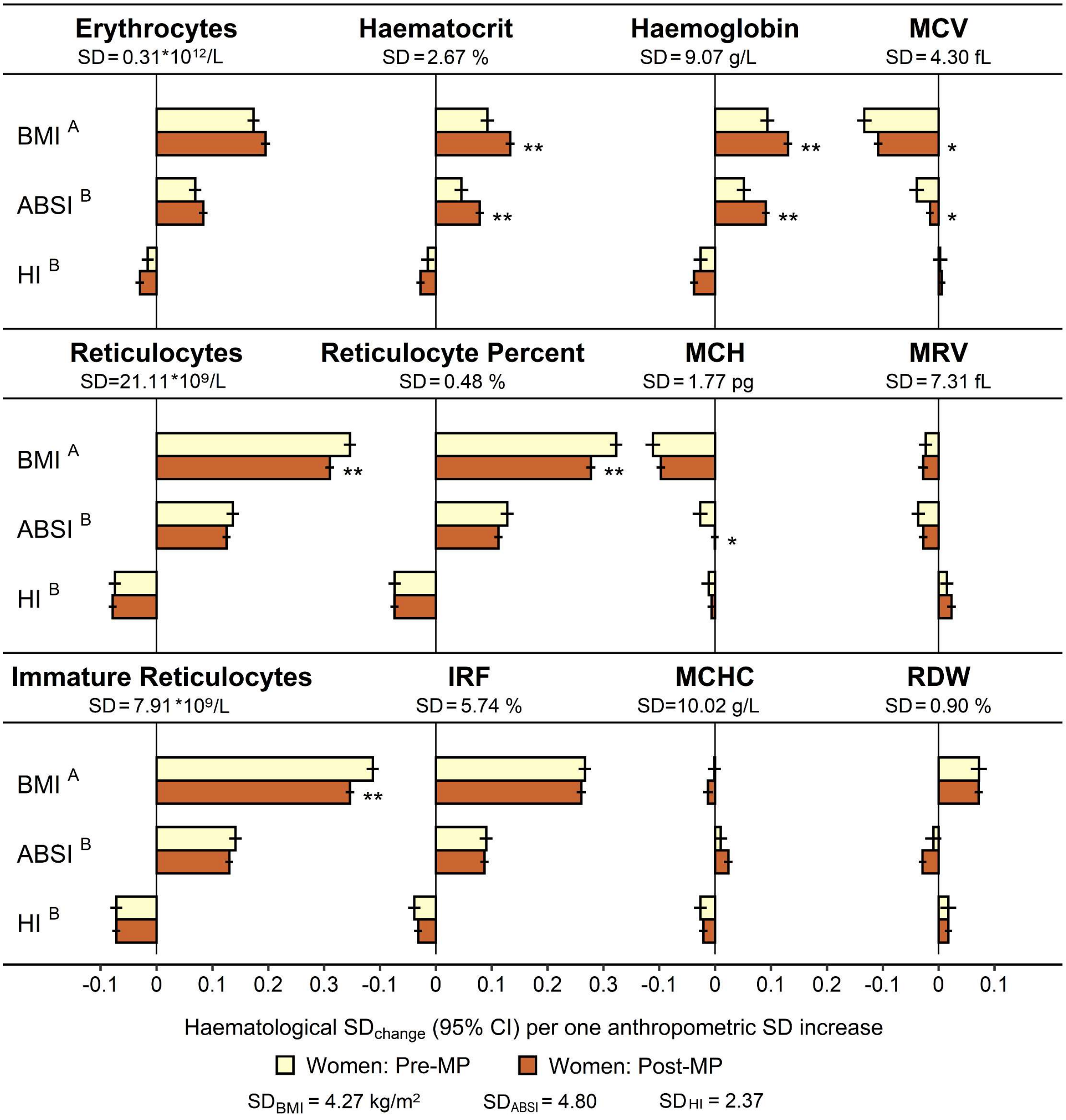

Supplementary Figure S1 Associations of obesity and body shape indices with erythrocyte and reticulocyte parameters according to menopausal status

ABSI – a body shape index; **BMI** – body mass index; **CI** – confidence interval; **HI** – hip index; **IRF** – immature reticulocyte fraction; **MCH** – mean corpuscular haemoglobin; **MCHC** – mean corpuscular haemoglobin concentration; **MCV** – mean corpuscular volume; **MRV** – mean reticulocyte volume; **Pre-MP** – pre-menopausal; **Post-MP** – post-menopausal; **RDW** – red cell distribution width; **SD** – standard deviation.

SD_{change} (95% CI) – regression coefficient (95% confidence interval) interpreted as the change in haematological parameters on SD scale per one SD increase of each anthropometric index. Estimates were obtained from multivariable linear regression models including each erythrocyte or

reticulocyte parameter as an outcome variable (sex-specific z-scores, value minus mean divided by SD). Exposure variables included BMI, ABSI, and HI (sex-specific z-scores). Adjustment variables include height, age, recent weight change, smoking status, alcohol consumption, physical activity, Townsend deprivation index, dietary intake (fruit, vegetables, red meat, processed meat, poultry, fish, cheese, bread, cereals, tea, coffee), recent major dietary change, region of the assessment centre, time of blood collection, fasting time, use of nonsteroidal anti-inflammatory drugs, use of vitamins ACE, vitamin D or minerals, multivitamins, hormonal replacement therapy use (Post-MP only), oral contraceptives use, and age at the last live birth.

[A] – p-value for the interaction term between BMI and menopausal status, from a joint model including Pre-MP (reference) and Post-MP women, with adjustment for ABSI, HI, and all covariates.

[B] – p-value for the interaction term between ABSI and menopausal status or HI and menopausal status, from a joint model including Pre-MP (reference) and Post-MP women, with adjustment for BMI and all covariates.

***** $p<0.001$; ****** $p<1*10^{-6}$

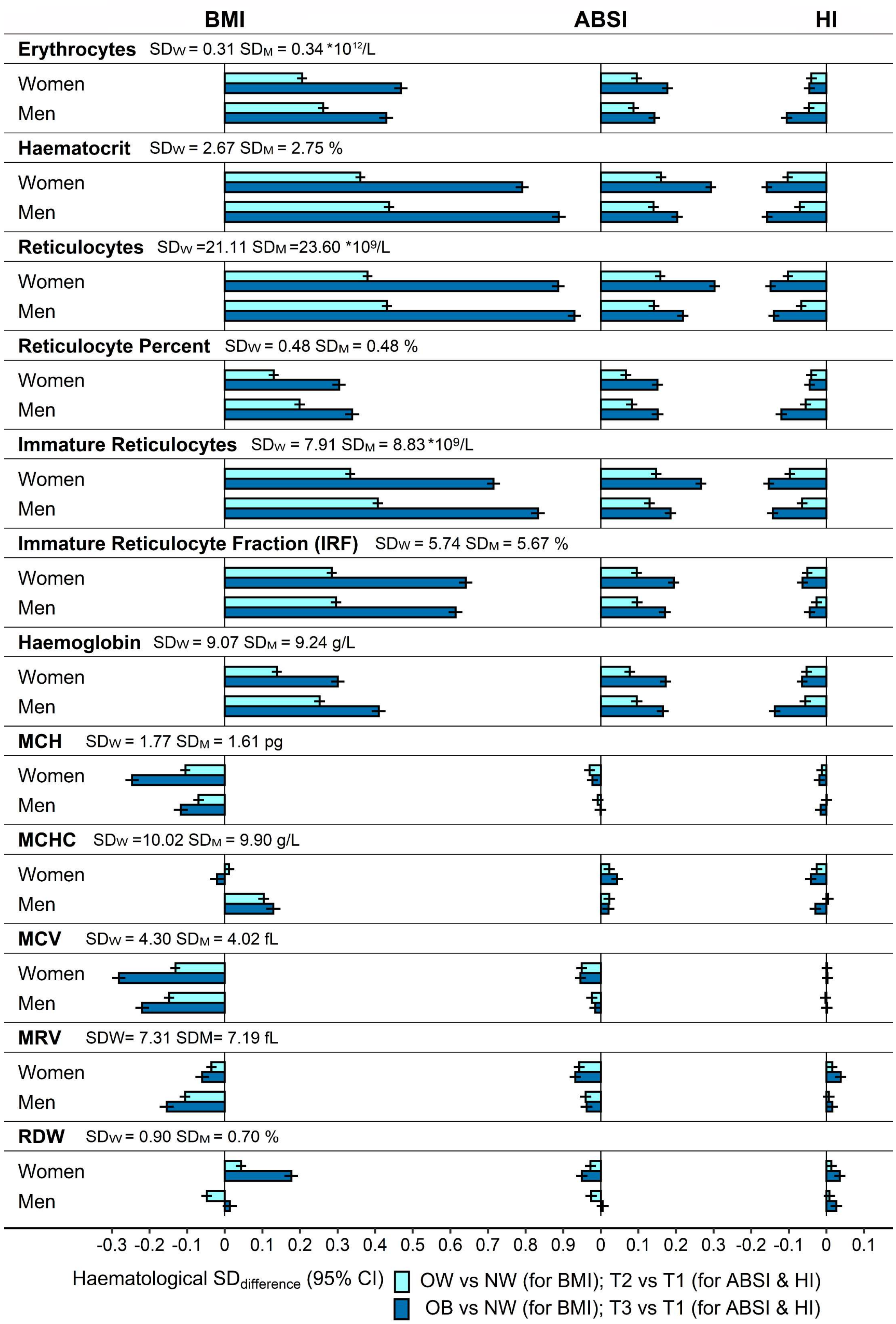

Supplementary Figure S2 Associations of obesity and body shape index categories with erythrocyte and reticulocyte parameters

ABSI – a body shape index (cut-offs: 71.038 and 75.055 in women; 77.467 and 80.868 in men); **BMI** – body mass index; **CI** – confidence interval; **HI** – hip index (cut-offs: 63.373 and 65.256 in women; 48.499 and 49.762 in men); **IRF** – immature reticulocyte fraction; **MCH** – mean corpuscular haemoglobin; **MCHC** – mean corpuscular haemoglobin concentration; **MCV** – mean corpuscular volume; **MRV** – mean reticulocyte volume; **NW** – normal weight (BMI≥18.5 to <25 kg/m^2); **OW** – overweight (BMI≥25 to <30 kg/m^2); **OB** – obese (BMI≥30 to <45 kg/m^2); **RDW** – red cell distribution width; **SD** – standard deviation (SD_W – women; SD_M – men); **T1-T3** – tertiles.

$SD_{difference}$ (95% CI) – regression coefficient (95% confidence interval) interpreted for each anthropometric category as the difference in a haematological parameter on SD scale compared to the reference category (NW for BMI, T1 for ABSI and HI). Estimates were obtained from multivariable linear regression models including each erythrocyte or reticulocyte parameter as an outcome variable (sex-specific z-scores, value minus mean divided by SD). Exposure variables included BMI categories, ABSI and HI tertiles. Adjustment variables included height, age, recent weight change, smoking status, alcohol consumption, physical activity, Townsend deprivation index, dietary intake (fruit, vegetables, red meat, processed meat, poultry, fish, cheese, bread, cereals, tea, coffee), recent major dietary change, region of the assessment centre, time of blood collection, fasting time, use of nonsteroidal anti-inflammatory drugs, use of vitamins ACE, vitamin D or minerals, and multivitamins, and for women, menopausal status, hormonal replacement therapy use, oral contraceptives use, and age at the last live birth.

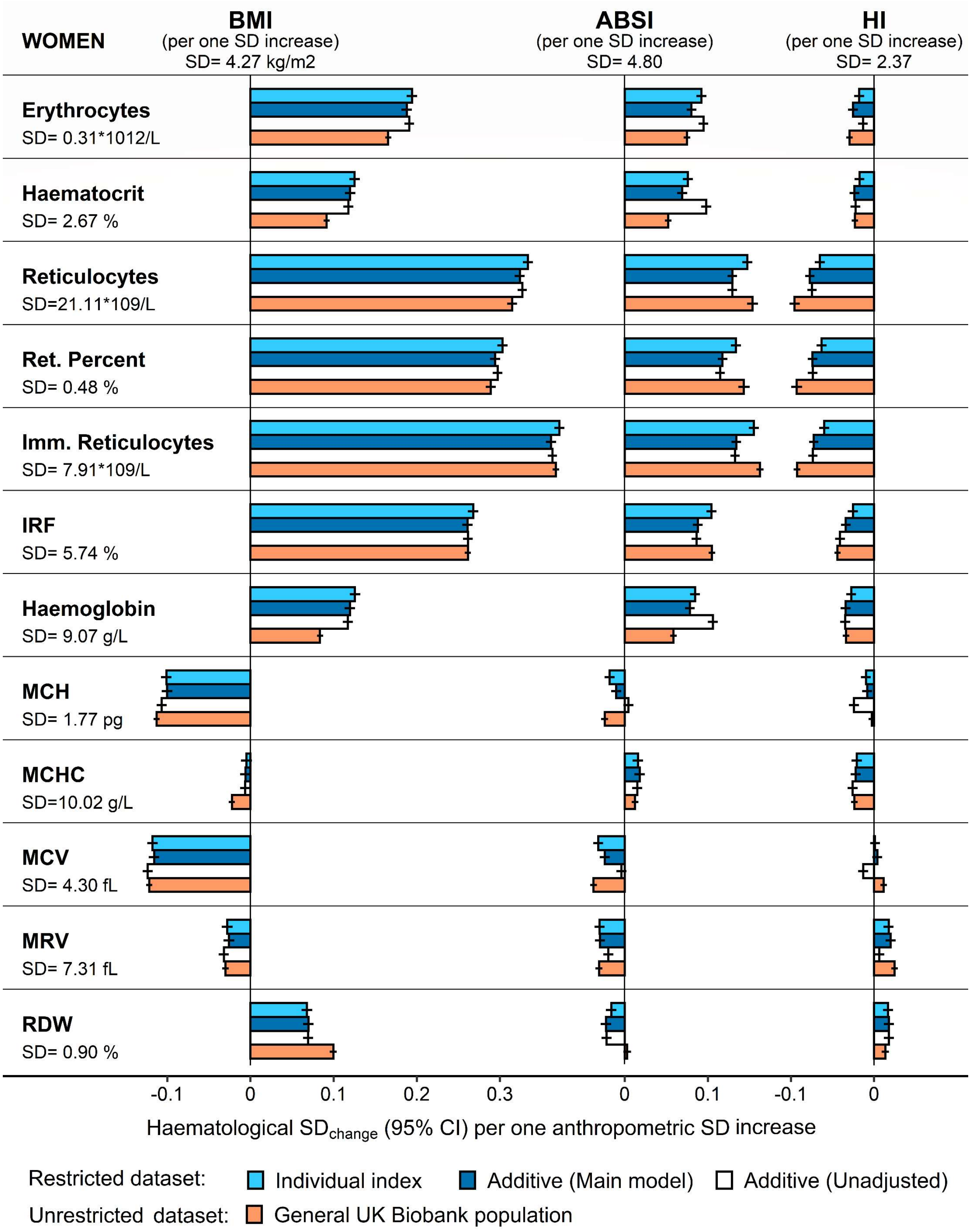

Supplementary Figure S3A Associations of obesity and body shape indices with erythrocyte and reticulocyte parameters: sensitivity analyses (women)

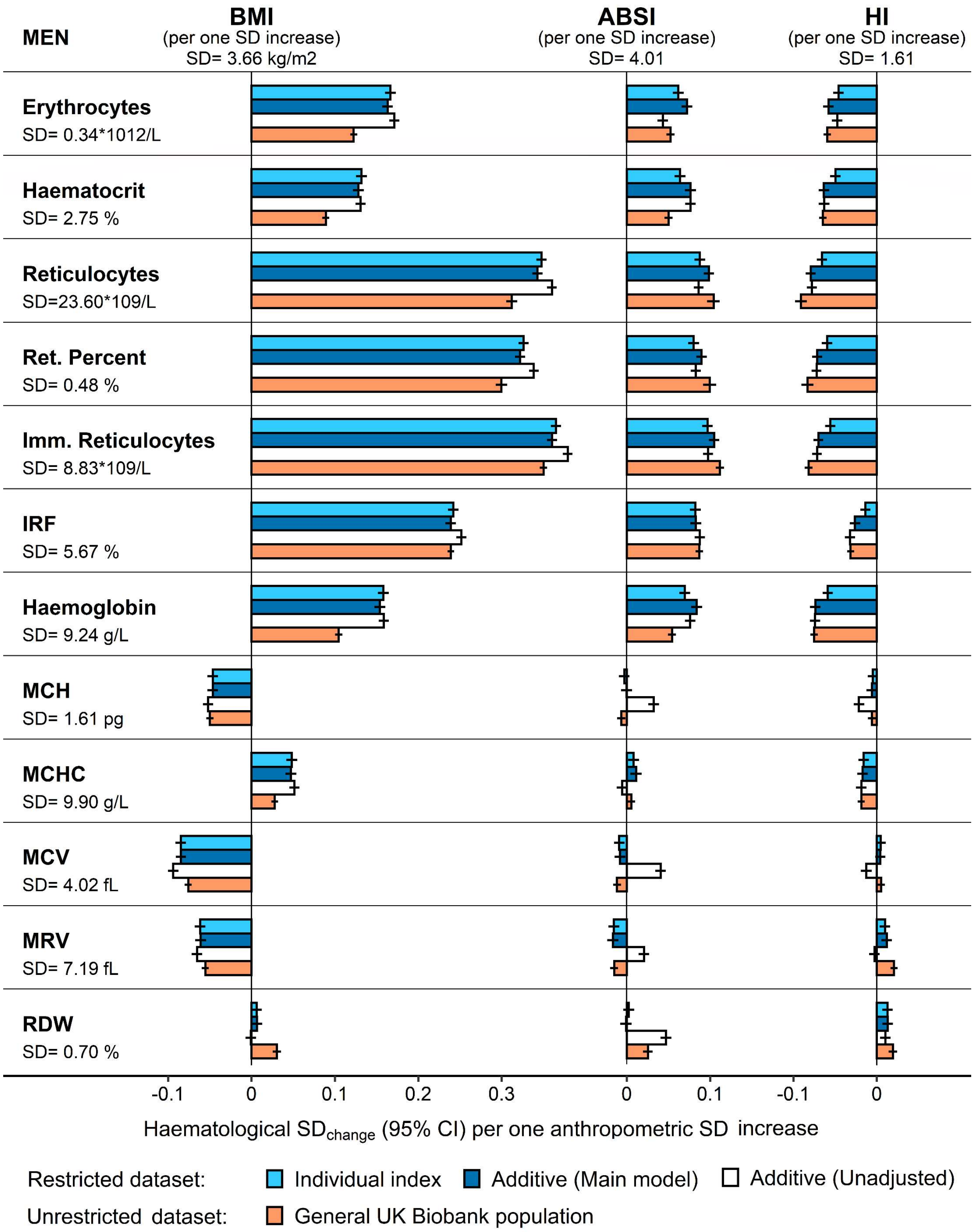

Supplementary Figure S3B Associations of obesity and body shape indices with erythrocyte and reticulocyte parameters: sensitivity analyses (men)

ABSI – a body shape index; **BMI** – body mass index; **CI** – confidence interval; **HI** – hip index; **IRF** – immature reticulocyte fraction; **MCH** – mean corpuscular haemoglobin; **MCHC** – mean corpuscular

haemoglobin concentration; **MCV** – mean corpuscular volume; **MRV** – mean reticulocyte volume; **RDW** – red cell distribution width; **SD** – standard deviation.

SD_{change} (95% CI) – regression coefficient (95% confidence interval) interpreted as the change in haematological parameters on SD scale per one SD increase of each anthropometric index. Estimates were obtained from multivariable linear regression models including each erythrocyte or reticulocyte parameter as an outcome variable (sex-specific z-scores, value minus mean divided by SD). All models were adjusted for the following covariates (unless otherwise specified): height, age, recent weight change, smoking status, alcohol consumption, physical activity, Townsend deprivation index, dietary intake (fruit, vegetables, red meat, processed meat, poultry, fish, cheese, bread, cereals, tea, coffee), recent major dietary change, region of the assessment centre, time of blood collection, fasting time, use of nonsteroidal anti-inflammatory drugs, use of vitamins ACE, vitamin D and minerals, or multivitamins, and for women, menopausal status, hormonal replacement therapy use (HRT), oral contraceptives use, and age at the last live birth.

Individual index – an adjusted model in the restricted dataset used in the main analysis (105,853 women; 100,854 men), with exposure variable one of BMI, ABSI, or HI (sex-specific z-scores), each in a separate model.

Additive (Main model) – an adjusted model in the restricted dataset, with exposure variables BMI, ABSI, and HI, all in the same model (shown in Figure 1).

Additive (Unadjusted) – a model in the restricted dataset, with exposure variables BMI, ABSI, and HI, all in the same model, without adjustment for covariates.

General UK Biobank population – a model in the unrestricted dataset with available anthropometric and haematological measurements, omitting all exclusions (253,218 women; 214,490 men), with exposure variables BMI, ABSI, and HI, all in the same model, and adjustment like the main model, but consolidating in a single category recent major dietary change due to illness or other reasons, and consolidating former and current use in a single category (ever use) for HRT use and oral contraceptives use.

References

Reference numbers correspond to the main document:

10. Christakoudi S, Tsilidis KK, Evangelou E, Riboli E. Association of body-shape phenotypes with imaging measures of body composition in the UK Biobank cohort: relevance to colon cancer risk. *BMC Cancer*. 2021;21(1):1106. doi:10.1186/s12885-021-08820-6.

13. Christakoudi, S., Riboli, E., Evangelou, E. & Tsilidis, K. K. Associations of body shape index (ABSI) and hip index with liver, metabolic, and inflammatory biomarkers in the UK Biobank cohort. *Sci Rep.* 2022;12(1):8812. doi:10.1038/s41598-022-12284-4.

16. Christakoudi, S., Tsilidis, K. K., Evangelou, E. & Riboli, E. A Body Shape Index (ABSI), hip index, and risk of cancer in the UK Biobank cohort. *Cancer Med.* 2021;10(16):5614-28; doi:10.1002/cam4.4097.

21. Christakoudi S, Tsilidis KK, Evangelou E, Riboli E. Sex differences in the associations of body size and body shape with platelets in the UK Biobank cohort. *Biol Sex Differ*. 2023;**14**(1):12. doi:10.1186/s13293-023-00494-y.
